# The impact of cancer therapy-related oral complications on the quality of life and well-being of childhood cancer survivors: A scoping review

**DOI:** 10.1371/journal.pone.0352194

**Published:** 2026-06-23

**Authors:** Nona Attaran Kakhki, Apoorva Sharma, Tara Balador, Franco A. Carnevale, Beatriz Ferraz Dos Santos, Mary Ellen Macdonald

**Affiliations:** 1 Faculty of Dental Medicine and Oral Health Sciences, McGill University, Montreal, Quebec, Canada; 2 Faculty of Art and Science, Concordia University, Montreal, Quebec, Canada; 3 Ingram School of Nursing, McGill University, Montreal, Quebec, Canada; 4 Faculty of Medicine, Dalhousie University, Halifax, Nova Scotia, Canada; University of Central Florida, UNITED STATES OF AMERICA

## Abstract

**Objective:**

This study aimed to review the scientific literature on the effects of cancer treatment-related oral complications on the quality of life and well-being of children surviving cancer. This study updates a previous review, from 2012, evaluating the oral health related quality of life and well-being of childhood cancer survivors and exploring the extent to which children’s perspectives are considered in research.

**Methods:**

We used a scoping review methodology informed by established frameworks: Arksey and O’Malley, Levac et al. and the Joanna Briggs Institute. Articles were retrieved from five electronic databases (MEDLINE/PubMed, Scopus, Embase, Web of Science, and PsychInfo) using a predefined search strategy. Screening and study selection were performed independently by two reviewers using Rayyan software (QCRI), with a third reviewer resolving disagreements. Reporting followed the PRISMA-ScR guidelines. Inclusion criteria included articles focused on the impact of oral complications on the quality of life and well-being of child cancer survivors (aged 0–17 years at the time of diagnosis), written in English or French, and published from 2012–2025. Exclusion criteria included articles involving non-childhood cancers, and with non-specific disease categories. The protocol for this review was published in *PLOS ONE* (https://doi.org/10.1371/journal.pone.0290364).

**Results:**

79 articles were included in this scoping review. The majority of studies had quantitative designs. In contrast to the original review, a substantial number of studies (47) reported children-reported symptoms and completed assessment tools rather than relying solely on their parents and health care providers to provide this information. Furthermore, since the original review, reporting of oral health-related quality of life (OHRQoL) measures has increased, however they were reported in a limited number of studies.

**Conclusion:**

Our review suggests that there is growing implementation of patient-reported outcomes and subjective measures of OHRQoL in assessing oral complications in pediatric cancer patients. Future studies should incorporate qualitative approaches to capture children’s or parents’ perspectives on cancer therapy, thereby complementing quantitative findings and providing a more comprehensive understanding of the multifaceted impact of oral complications on children’s quality of life.

## Introduction

According to the World Health Organization, in 2020 nearly 280 000 children and young adults around the world were diagnosed with cancer, of which approximately 110 000 died [1]. In Canada, an estimated 4,975 children were newly diagnosed with cancer between 2018 and 2023 [2]. Despite persistent and substantial disparities across cancer types and resource settings, advances in treatment modalities, such as chemotherapeutic agents, have contributed to a general worldwide decline in pediatric cancer mortality rates [3]. Childhood cancer survival rates vary drastically by country, exceeding 80–90% in high-income nations (e.g., Canada, USA, Finland) while falling below 30–40% in many low- and middle-income countries. The 5-year survival rate for acute lymphoblastic leukemia can range from over 90% in high-income areas to as low as 30% in some African countries [4]. Moreover, the survival rate of cancer in pediatric patients depends on factors such as the type of cancer, cellular changes, assigned treatment, and the general health of the patient [5]. The most common pediatric malignancies are leukemia, brain tumor, lymphoma, Wilms’ tumor, and neuroblastoma [1]. Different types of cancer and their associated risk factors require different therapeutic approaches [6]. Frequently used treatment modalities include surgery, chemotherapy, immunotherapy, radiation therapy, and stem cell transplant [7]. Chemotherapy and radiation therapy function by destroying fast growing, rapidly dividing cancerous cells; however, they do not differentiate between healthy and cancerous cells, causing side effects [7]. Due to the high turnover rate of cells in the oral mucosa, oral cavity is a common location for such side effects – called ‘oral complications’ – including the soft and hard tissues in the mouth [8,9]. The occurrence and severity of these oral complications vary according to the type of cancer, child’s age, their oral health status at the time of diagnosis, and the type and dose of oncologic treatments administered [6,10,11].

Treatment-related oral complications can occur during or soon after treatment, or months – even years – later. They are classified as early (acute) effects and late (chronic) effects [12]. Early oral effects include oral mucositis, xerostomia (dry mouth), oral infections (e.g., candidiasis and herpes virus infections), and taste disturbances. Late effects include dental decay, and abnormalities in dental and jaw development. Combined treatments such as combination of chemotherapy with radiation increase the risk of dental problems such as dental caries, taste disturbances, and missing teeth or roots [9]. These complications can be particularly difficult for children who have undergone cancer therapy and they can influence their physical, functional, and psychosocial health, months or years after the treatment is completed [13].

In 2012, a scoping review was conducted by Noronha and Macdonald to review published literature on the impact of cancer therapy-related oral complications on the quality of life of pediatric patients [13]**.** Since this review, the oral health-specific measure, oral health-related quality of life (OHRQoL), has become more commonly used in studies assessing the effects of oral diseases and oral complications on patients’ quality of life. This multidimensional construct focuses on how an individual’s oral health affects their comfort, abilities and well-being (e.g., eating, sleeping, social interactions, self-esteem) [14]. The increased use of OHRQoL follows the growing recognition of oral health as an essential component of systemic health and general well-being [15,16]. As oral health is strongly age-dependent, and therefore OHRQoL in children is different from adults, this measure has been adapted for child populations [17].

Further, this prior review found a dearth of qualitative research into the experiences of children about how oral side effects of cancer therapy impacts their quality of life [13]. This result is not surprising; a 2007 review of pediatric oncology studies, by Hinds et al highlighted the value of child-focused patient reported outcomes in children with cancer at the end of life. This review showed that 85% of studies on childhood cancer conducted between 2002 and 2006 did not solicit patient-reported outcomes, instead relying on parent and health care professional’s reports which were insufficient to capture the child’s experiences and personal preference [18]. While having the parents’ and health care providers’ perspective is clearly important, an empirical finding by Eiser et al in 2013 showed discordance between child and adult-reported outcomes, highlighting that children’s perspectives are not always consistent with adults [19]. As a result, a new approach to child-focused research by Carnevale et al in 2020 has started to engage children directly in research to better understand their experiences firsthand [20]. This movement is consistent with article 12 of The United Nations Convention on the Rights of the Child which stipulates that children’s experiences must be rendered through their own voices and that they have a right to express their own views in matters that affect them [21]. While dental research has started to follow this guiding principle [22], it is not known if or how research on the impact of cancer treatment on children’s quality of life and well-being has followed suit since Noronha and Macdonald’s review published in 2012, which covered studies up to 2011 [23].

We therefore updated this review with the following additions: we aimed to first, assess whether (and how) there has been increased attention to OHRQoL; second, we expanded the scope of the study to include adult survivors of childhood cancer to capture the long-term impact of cancer treatment complications on quality of life and well-being; and third, we were especially interested to see if and how research approaches increasingly acknowledge and incorporate the unique insights, experiences, and perspectives of children surviving cancer on their quality of life and well-being.

## Materials and methods

### Protocol and registration

A protocol of this scoping review has been published (https://doi.org/10.1371/journal.pone.0290364) [24].

### Study design

The scoping review followed the Joanna Briggs Institute Reviewer’s Manual to assure transparency, accuracy, and completeness [25] and the Arksey and O’Malley’s methodological framework of six stages for conducting a scoping review, with Levac et al’s additions to the framework [26,27].

#### Identifying the research question.

Our primary research question was: What are the impacts of oral complications from cancer therapy on the quality of life and well-being of childhood cancer survivors?

And our secondary research question was: How are children involved in producing knowledge related to the effects of cancer treatment on their quality of life and well-being?

#### Identifying relevant studies.

The identification of relevant literature consisted of several combined approaches, including searching electronic databases, grey literature sources, and screening reference lists. Studies were included if they examined the impact of oral complications on the quality of life and well-being of childhood cancer survivors (aged 0–17 years at diagnosis), were published in English or French, and were published between 2012 and 2025. Studies focusing on non-childhood cancers or non-specific disease categories were excluded

Articles were accessed through five electronic databases: MEDLINE/PubMed, Scopus, Embase, Web of Science, and PsychInfo. A librarian (MM) created the search strategy, led citation management, and assisted with search documentation. (Table 1) All databases were searched, and the reference lists of articles included were manually screened to search for other relevant studies. The initial search strategy was piloted to ensure breadth, comprehensiveness, and feasibility and was subsequently adapted for each database.

**Table:** 

Search Strategy
1. exp Antineoplastic Agents/ 2. exp Radiotherapy/ 3. exp Hematopoietic Stem Cell Transplantation/ 4. exp Bone Marrow Transplantation/ 5. (antineoplastic or chemotherap* or radiotherap* or ((h?ematopoietic or bone marrow) adj3 (SCT or transplant*))).tw,kw. 6. or/1–5 7. Oral Health/ or exp Dentistry/ or Halitosis/ or exp Stomatognathic Diseases/ or DMF Index/ or Periodontal Index/ 8. (dentist* or endodont* or orthodonti* or periodont* or prosthodont* or apicoectom* or gingivectom* or gingivoplast* or glossectom* or “mandibular advancement” or alveolectom* or alveoloplast* or vestibuloplast* or “root canal” or (oral adj1 (care or health or hygiene or surgical or surgery or mucositis)) or oropharyng* or temporomandibular or TMJ or jaw or jaws or mandibular or maxillofacial or mandible* or maxilla* or “alveolar ridge” or dental or orthognathic or tooth or teeth or occlusion or malocclusion or mal-occlusion or odontolog* or tongue* or glossal or buccal or palatal or palate or palates or labial or lip or lips or gingiva* or gingiviti* or saliva* or DMF).tw,kw. 9. 7 or 8 10. “Quality of Life”/ or exp rehabilitation/ or exp eating/ or exp human activities/ or (rh or px).fs. 11. (quality of life or well-being or long-term or (daily adj1 (life or living)) or rehabilitat* or depress* or pain or immunosuppress* or “disease management” or “Child Oral Health Impact Profile” or C-DAS or CFSS-DS or COHRQoL or COHIP or CPQ or ECOHIS or FIS or OASIS or OHQoL or OHRQoL or QOL or P-CPQ or POQL or ((“Early Childhood Oral Health Impact” or “Oral Aesthetic Subjective Impact” or “Corah Dental Anxiety” or “Family Impact”) adj1 Scale) or “Children’s Fear Survey Schedule” or ((“Child” or “Parental-Caregiver”) adj1 “Perceptions Questionnaire”)).tw,kw. 12. 10 or 11 13. 6 and 9 and 12 14. limit 13 to “all child (0–17 years)” 15. exp Child/ or exp Pediatrics/ 16. (infan* or toddler* or minors or boy? or boyhood or girl? or child* or schoolchild* or school child* or adolescen* or juvenil* or youth* or teen* or under*age* or p?ediatric*).tw,kf. 17. 15 or 16 18. 13 and 17 19. 14 or 18 20. limit 19 to (english or french)

#### Selecting studies.

All retained studies were merged into a single EndNote library, with duplicates removed. The library was then imported into Rayyan software (QCRI) for screening. Two reviewers (AS and NA) independently screened the titles and abstracts of the first 50 studies, calibrated their assessments based on inclusion and exclusion criteria, and measured inter-rater reliability using Cohen’s kappa coefficient. In cases of low agreement (<0.40), the reviewers discussed discrepancies and refined the eligibility criteria to improve clarity and consistency. This process was repeated until substantial agreement was achieved (>0.40). The final agreed-upon criteria were then applied uniformly to the screening of all remaining studies.

The screening and selection process consisted of two steps, starting with title and abstract screening and then full-text review of studies deemed potentially eligible. In both steps, reviewers independently recorded reasons for inclusion or exclusion in Rayyan and resolved disagreements through discussion or consultation with a third reviewer (MEM).

#### Charting the data.

A data extraction tool was developed, including author, publication year, location of the study, study design, age of children, type of cancer, type of treatment, type of oral complications, OHRQoL measurement tools and assessment strategies, the type of children’s involvement in the study, and findings related to the effects of cancer treatment on the OHRQoL of children (S1 Table). To ensure that all relevant data were extracted, the tool was piloted by the two reviewers on 10 articles prior to implementation. Differences in data extraction were then discussed and the tool refined.

In the charting phase, reviewers periodically compared extracted data. Inconsistencies and disagreements were discussed and a senior researcher (MEM) resolved disputes. The tool was iteratively updated as required during the extraction process. Throughout the process, weekly team meetings were held during which ambiguities, concerns, or other issues were discussed.

#### Collating, summarizing, and reporting findings.

**Fig 1:**
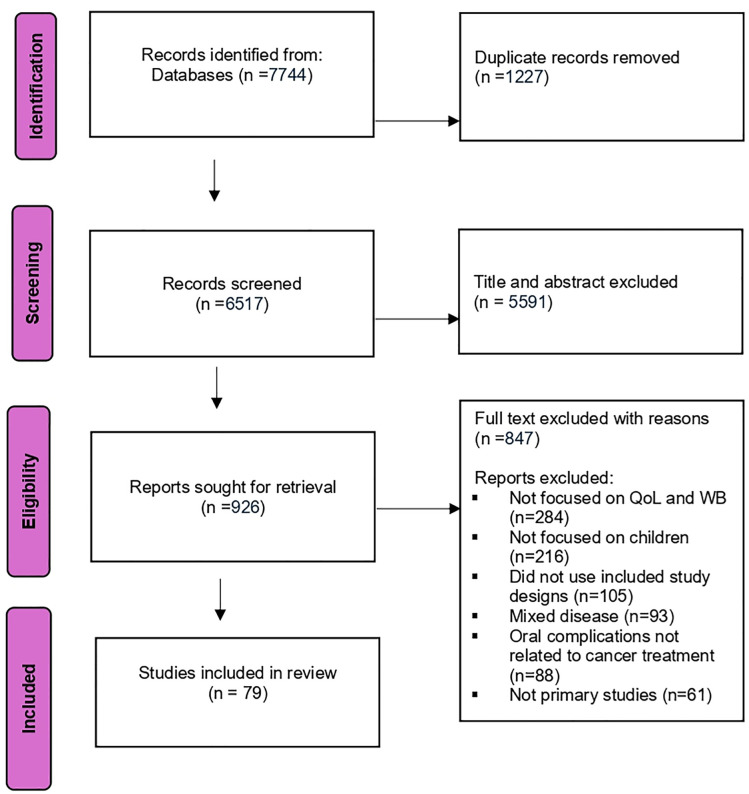
PRISMA flow diagram.

Given the goal to present a comprehensive summary of existing evidence and significant findings across various domains, the chosen analytical approach was descriptive and narrative. The following three steps were followed [27]: First, the data from the review process and findings were reported following the PRISMA-ScR framework (The Preferred Reporting Items for Systematic Reviews and Meta-Analyses extension for Scoping Reviews; see Fig 1), which is designed to ensure transparent, comprehensive, and standardized reporting of scoping review methodology and results. Second, the data were analyzed specifically regarding the impact of cancer therapy on the quality of life and well-being of children and their involvement in producing this knowledge in studies. Graphs, charts, or tables were used where relevant, with an accompanying narrative summary. Finally, knowledge gaps and broader implications for future research, policy, and practice were identified. This stage contributed to a comprehensive understanding of the current state of the literature and provided direction for advancing research and practice in this field.

## Results

Our final sample was comprised of 79 articles (Fig 1). The Inter-rater reliability between two reviewers, using Cohen’s kappa coefficient was 0.79, which indicates substantial agreement between reviewers.

### Data demography

Of the 79 retained articles, 50 used a quantitative design; in addition, there were 23 case reports, 3 case series [28–30] and 2 clinical reports [31,32]. Only one study had a qualitative design [33].

A total of 41 pediatric cancer types were included in the studies. The most prevalent reported malignancies were leukemia (including acute lymphoblastic leukemia, acute myeloid leukemia), lymphoma (including Hodgkin’s lymphoma, non-Hodgkin’s lymphoma, Burkitt lymphoma), sarcomas (including Rhabdomyosarcoma, and osteosarcoma), and central nervous system (CNS) solid tumors, and neuroblastoma. Other malignant conditions included Retinoblastoma, Nasopharyngeal carcinoma, stem cell glioma, among others. Seventy-one articles involved chemotherapy with or without other cancer treatments. Oral mucositis was reported as the most prevalent oral complication as well as the most prevalent early oral complication across the reviewed articles, with 24 publications focusing exclusively on it. Other early oral complications included taste change, hyposalivation, cracked lips, infections, gingivitis. More common late oral complications reported were dental caries in 16 articles, tooth and jaw abnormalities in 11 articles, trismus in 7 articles and tooth discoloration in 3 articles (S1 Table).

### Quality of life and well-being

**Fig 2:**
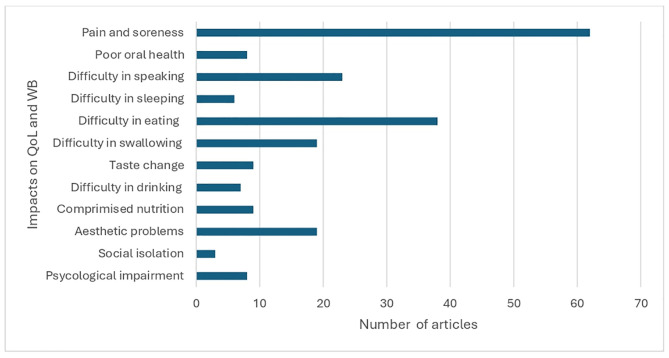
Distribution of articles addressing the impact of oral complications on quality of life and well-being.

While not all articles explicitly focused on quality of life and well-being, all 79 studies reported short- or long-term oral complications resulting from cancer therapies, with implications for children’s physical, psychological, and social health (S1 Table). A wide range of these complications were identified, including oral pain in 62 article, difficulty with swallowing in 19 article, eating in 38 article, speaking in 23 article, and sleeping in 6 article, as well as reduced enjoyment of food in 2 article. Additional impacts included emotional distress, compromised nutrition in 9 article, impaired speech, altered taste perception in 9 article, psychosocial challenges in 8 article, poor self-esteem and social isolation in 3 article, and inadequate oral hygiene in 8 article (Fig 2).

Among these complications, pain was the most frequently reported consequence, documented in 62 studies (Fig 2). Reports of pain spanned various regions, including the oral cavity, jaw, throat, and teeth, highlighting its pervasive impact on multiple dimensions of well-being.

The findings indicate that difficulty in swallowing, eating, speaking, and sleeping primarily affects functional well-being, often leading to frustration and emotional distress in children. Compromised nutrition and poor oral hygiene were classified as health-related consequences, significantly influencing children’s overall quality of life. Furthermore, aesthetic concerns, speech difficulties, and social discomfort contributed to social isolation, reinforcing the psychological burden associated with oral health complications.

A minority of studies included a specific QoL (3 articles) [34–36] or OHRQoL measure (17 articles). The only QoL measure was PedsQL™ 3.0 (Pediatric Quality of Life Inventory TM) module which is a tool used to evaluate the overall QoL in the pediatric population. While this measure is not specific to oral health, the results were connected to oral health. For example, the findings of one study [34] indicated that cognitive difficulties and smiling with embarrassment were two factors linked to impaired QoL. In addition, studies involving OHRQoL measures aimed to identify the specific oral health factors that affect QoL. For instance, one study [37] using OMQoL (Oral Mucositis Quality of Life) scores revealed that difficulties swallowing and sleeping had the strong influence on OHRQoL. These factors were also described as contributing to lower QoL and higher distress levels. (S1 Table)

OHRQoL measurement tools included OHIP-14 (Oral Health Impact Profile-14), Oral Mucositis Daily Questionnaire (OMDQ), OMQoL questionnaire, ChIMES (Children’s International Mucositis Evaluation Scale), Early Childhood Oral Health Impact Scale (ECOHIST) and Child Perception Questionnaire (CPQ), Child Oral Health Impact Profile score (COHIP) and Child-Oral Impacts on Daily Performances (Child-OIDP). (Table 2)

**Table 2:** Oral health related quality of life measurement tools included in the literature.

Type of measurement	Number of articles	References
**OHIP-14**	4	Bardellini, E [38], Shum, M [39], Stolze, J [40], Bresolin, CR [41]
**OMDQ**	3	Manji, A [42], Cheng, K [37], Cheng, K [43]
**OMQoL**	2	Cheng, K [37], Ip, WY [44]
**ChIMES**	2	Kostak, M [45], Khurana, H [46]
**ECOHIS-T**	2	Klik, M [47], Bresolin, CR [41]
**CPQ**	2	Okello, D [48], Bresolin, CR [41]
**COHIP**	1	AlShamali, S [49]
**Child-OIDP**	1	Bensouda, S [50]

### Involvement of children

Among the articles examined, 52 articles provided information regarding children’s involvement in the research, but the amount of children’s involvement was different. Notably, in 47 articles participants themselves reported their symptoms and completed the questionnaires or contributed to interviews independently and in 5 studies [33,35,37,43,51] it was indicated that the caregiver, health care provider, or both assisted the participant to report if needed.

### Comparison between the original review and our study

We sought to identify similarities and differences between the original review and our study. (Table 3)

**Table 3:** Overview of key findings from the original review (inclusive of years 2000-2011) and current scoping review (inclusive of years 2012-2025).

Characteristic	n (%) of studies in original review (Total studies=82)	n (%) of studies in Current Review (Total studies=79)
**Study design**		
Quantitative studies	78 (95.1)	49 (62.0)
Case reports	Not Reported	23 (29.1)
Case series	Not Reported	3 (3.8)
Clinical reports	Not Reported	2 (2.5)
Qualitative studies	3 (3.7)	1 (1.3)
Mixed-methods studies	1 (1.2)	1 (1.3)
**Cancer type included**		
Leukemia	69 (84.1)	77 (53.1)
CNS tumors	Not Reported	15 (10.3)
Solid tumors (incl. sarcoma, neuroblastoma, Wilms tumor)	Not Reported	38 (26.2)
Other tumors	Not Reported	15 (10.3)
**Cancer treatment modalities included**		
Chemotherapy	80 (97.5)	74 (90.0)
Radiotherapy	11 (13.4)	31 (39.2)
HSCT	14 (17.0)	18 (22.7)
Surgery	Not Reported	16 (20.2)
**Oral complications**		
Oral mucositis	39 (47.5)	41(51.8)
Dental caries	7 (8.5)	17 (21.5)
Xerostomia / salivary changes	6 (7.3)	14 (17.7)
Dental developmental anomalies	17 (20.7)	14 (17.7)
Other complications	13 (15.8)	7 (8.8)
**OHRQoL assessment**		
Oral health related Quality of life and well-being impact reported	21 (25.0)	79 (100)
Use of validated tools	Not Reported	17 (21.5)
**Children’s involvement in outcome reporting**		
Child participating	3 (3.6)	47 (59.5)

In terms of the impact on quality of life and well-being, both reviews observed adverse effects that affected participants’ functional, physical, psychological, and social well-being. The original review included 21 articles on QoL impacts, while our review identified 79 publications on this topic. The original review did not explore specific measures of QoL or OHRQoL; in contrast, our review noted 16 studies that used QoL measures (3 articles) and OHRQoL measures (13 articles). In terms of symptom reporting, the previous review indicated that caregivers were most involved in reporting symptoms (3 articles), whereas our findings revealed that participants themselves self-reported symptoms or completed surveys, questionnaires, or interviews (47 articles). Moreover, our review incorporates 6 studies featuring adult participants with a history of childhood cancer, which were not included in the original review.

## Discussion

This scoping review provides an updated synthesis of literature examining the impact of cancer treatment-related oral complications on the quality of life and well-being of childhood cancer survivors. In relation to the previous review by Noronha and Macdonald [13], our findings highlight notable changes in research approaches, particularly in the increased inclusion of patient-reported outcomes and the use of validated QoL and OHRQoL measurement tools. Despite these changes, gaps remain in fully capturing the multidimensional impact of oral complications on children’s overall well-being.

One of the most notable shifts observed in the literature since the original review is the shift toward incorporating children’s perspectives in research. While Noronha and Macdonald reported that only three studies included direct child self-reports, our review identified 47 studies in which children actively reported their symptoms, either independently or with caregiver assistance. This change aligns with the broader movement toward patient-centered research, following the concern that children’s lived experiences may differ from caregiver or healthcare provider reports. This is particularly relevant in assessing subjective factors such as pain, emotional distress, and social functioning, which may be underrepresented when reported by proxies [52].

Interestingly, while children’s direct participation has increased, this shift has not been accompanied by a comparable rise in qualitative study designs. Instead, greater child involvement has primarily occurred through the expanded use of standardized QoL and OHRQoL measurement tools. Questionnaire-based approaches are often perceived as more feasible, time-efficient, and ethically manageable in clinical research involving children. Standardized tools allow children’s perspectives to be captured in a structured way while minimizing methodological, ethical, and logistical challenges associated with conducting in-depth qualitative interviews, particularly in vulnerable pediatric populations. However, it remains unclear whether this shift has yielded substantially different or more nuanced understandings of children’s lived experiences. Thus, while the increasing use of QoL instruments represents meaningful progress toward amplifying children’s voices, it also highlights an ongoing gap in qualitative exploration of children’s lived experiences.

In line with this trend, the increased adoption of OHRQoL tools, such as the Child Perception Questionnaire (CPQ), the Oral Health Impact Profile (OHIP-14), and the Oral Mucositis Quality of Life questionnaire (OMQoL), demonstrates a growing recognition of the need for standardized measures to assess the impact of oral health complications. These tools provide a structured approach to evaluating functional, emotional, and social domains affected by oral complications [53], though their use remains relatively limited and not specifically for children surviving cancer. Consequently, future research would benefit from both the refinement of existing measurement tools and the integration of i in-depth qualitative methodologies to accurately capture the lived experiences of children undergoing cancer treatment. Exploring their perspectives, emotions, and challenges in detail will enhance the relevance and effectiveness of future QoL measurement instruments and approaches, ensuring they reflect the unique experiences of this vulnerable population.

The reviewed studies collectively indicate that oral complications significantly disrupt various aspects of children’s daily lives. The most reported issues included oral pain (reported in 62 studies), difficulty swallowing, speaking, and eating, all of which contribute to distress and compromised nutrition. Furthermore, psychosocial impacts such as embarrassment, reduced self-esteem, and social withdrawal were frequently observed, particularly among children experiencing dental anomalies or disfigurement [54].

Notably, our findings suggest that while early complications like oral mucositis are well-documented, long-term consequences of cancer therapy, such as dental developmental anomalies, and trismus remain underexplored. These late effects can persist into adulthood, influencing survivors’ QoL long after cancer remission. Studies involving adult survivors of childhood cancer have begun to shed light on these long-term challenges [55], along with a wide range of non-oral late effects, including cardiovascular complications, endocrine and growth disorders, neurocognitive impairment, chronic fatigue, and psychosocial difficulties such as anxiety, depression, and challenges with social adjustment [56]. Longitudinal research is needed to comprehensively understand their trajectory and to inform the development of targeted interventions.

This review underscores the complex and multifaceted impact of oral complications from cancer therapy on children, with potential implications for clinical practice. These results highlight the need for interdisciplinary care approaches that go beyond managing acute symptoms. Pediatric oncology teams, including dentists, nurses, psychologists, and nutritionists, should ensure that oral health assessments are systematically integrated and standardized across care pathways, as routine dental care enables the early identification of potential acute and late oral complications and supports the timely initiation of appropriate preventive and therapeutic interventions [57,58]. Additionally, psychosocial support should be integrated into care plans, particularly for children who experience visible dental anomalies or treatment-related disfigurement, as these may contribute to reduced self-esteem and social withdrawal [59]. These findings call for the development of personalized interventions that address each child’s specific physical, emotional, and social needs, aiming to improve both immediate comfort and long-term quality of life.

Moreover, given the complex and subjective nature of QoL experiences, future studies should adopt age-appropriate participatory research methods, taking into account children’s communication abilities and ethical considerations to capture the nuanced perspectives of childhood cancer survivors. At a policy and health-system level, these findings support the need for greater integration of oral health within pediatric oncology care pathways. In the Canadian context, this could include the development of national or provincial guidelines mandating standardized oral health screening before, during, and after cancer treatment, as well as the systematic inclusion of OHRQoL indicators within survivorship care plans. Incorporating oral health metrics into electronic health records and oncology registries would facilitate longitudinal monitoring and improve data consistency across institutions. Furthermore, strengthening collaboration between pediatric oncology services and dental care providers through shared protocols and referral pathways could enhance continuity of care and early intervention for oral complications.

As with all studies, this review has limitations, one of which is that only studies published in English and French were included; we thus may have excluded relevant research published in other languages. Further, we did not assess the methodological quality of included articles. Additionally, given the specific aims of this review, we did not conduct analyses based on country of origin. As a result, potential cultural, healthcare system, and contextual differences across settings were not examined, which may limit the generalizability of the findings to diverse populations and contexts.

## Conclusion

In the last 13 years since the original review, the literature on the impact of cancer therapy-related oral complications on the quality of life and well-being of pediatric cancer survivors has evolved. A key development is the increased use of QoL and OHRQoL measures. Additionally, there has been a shift towards including children-reported symptoms rather than relying solely on parental or healthcare provider assessments. Future studies should incorporate QoL explicitly to provide insight into the impact of cancer treatment on children’s well-being and oral health. Moreover, integrating these measures alongside qualitative research methods can help understand children’s subjective lived experiences during cancer therapy. By delving deeper into children’s own perspectives, emotions, and challenges, researchers may gain a more nuanced and specific understanding of how these oral complications affect their overall quality of life and well-being. Combining these approaches, particularly within longitudinal research designs, can support a more comprehensive understanding of children’s evolving needs over time and inform the development of targeted and supportive interventions, thereby improving the overall well-being and survivorship experience of pediatric cancer patients.

## Supporting information

S1 Table Impact of cancer treatment on quality of life and well-being of children and their involvement in the studies. (DOCX)

S2 Checklist Prisma-ScR checklist. (DOCX)
