# Supplementary material for: The impact of cancer therapy-related oral complications on the quality of life and well-being of childhood cancer survivors: A scoping review
Source: PLoS One. 2026 Jun 23;21(6):e0352194. doi: 10.1371/journal.pone.0352194 (PMC13289919; doi:10.1371/journal.pone.0352194)
Supplement: S1 Table — (DOCX) [file pone.0352194.s001.docx]

**S1 Table:** [**Impact of cancer treatment on QoL and well-being of children and their involvement in the studies**](#_Toc194889025)

|  | **First Author**  **(S1 Table ref #)** | **Corresponding main text citation** | **Age at the time of study** | **Type of cancer** | **Treatment** | **Oral Complication** | **Oral Health**  **and QoL Impact** | **Measurement tool** | **Children involvement** |
| --- | --- | --- | --- | --- | --- | --- | --- | --- | --- |
| **1** | **Agholme, M. B. (1)** | **—** | **10.2 (7.5–12.5)** | **leukemia** | **HSCT** | **Blisters and oral ulcerations, including mucositis** | **Severe pain**  **Discomfortability**  **Impact on daily functioning** | **- WHO mucositis scale**  **Questionnaire - Self-report subjective complaints through bedside interviews.** | **The questionnaire was self reported and children answered the questions** |
| **2** | **Alonso-Prieto,**  **M. (2)** | **—** | **4, 17** | **Acute myeloid leukemia, acute lymphoid leukemia** | **CT, HSCT** | **Oral mucositis** | **Oral pain** | **WHO mucositis scale**  **Numerical Rating Scale (score 0-10) to measure pain intensity** | **Participant self-reported the symptoms** |
| **3** | **Amadori, F. (3)** | **—** | **3-18** | **Leukemia, lymphoma solid tumours** | **CT** | **Oral mucositis** | **Oral pain** | **VAS for pain**  **WHO mucositis scale** | **Participants self-reported the symptoms**  **Participants and their**  **caregiver gave consent** |
| **4** | **Arpaci, T. (4)** | **—** | **3-18** | **Central nervous system tumor, leukemia,**  **solid tumor, lymphoma, others** | **CT** | **Oral mucositis, dysgeusia** | **Poor nutrition, taste changes, oral pain** |  | **Caregivers perspective was reported** |
| **5** | **Attinà, G. (5)** | **—** | **4-18** | **Central nervous system tumor, sarcoma, lymphoma, others solid tumors** | **CT** | **Oral mucositis, ulcers** | **Oral pain, difficulty eating, nutritional support, poor**  **QoL** | **WHO mucositis scale**  **Wong–Baker FACES Pain Rating Scale (WBS)** | **Caregiver gave consent** |
| **6** | **Bardellini, E. (6)** | **38** | **6-14** | **Acute lymphoid leukemia** | **CT** | **Oral mucositis** | **Oral pain, poor QoL** | **Short form of Oral Health Impact Profile (OHIP-14)**  **questionnaires**  **7Common Toxicity C8riteria (CTC) scale of 9the WHO** | **Participants answered the survey**  **Participants and their caregiver gave consent** |
| **7** | **Bardellini, E. (7)** | **—** | **5-18** | **Acute lymphoid leukemia** | **CT** | **Oral mucositis** | **Oral pain, difficulty eating, swallowing and speaking** | **VAS10 for pain CTC scale11** | **Participants and their caregivers gave consent** |
| **8** | **Bardellini, E. (8)** | **—** | **16** | **Burkitt lymphoma** | **CT** | **Oral mucositis, ulcers, bacterial infection** | **Oral pain, difficulty in chewing and swallowing** | **WHO m12ucositis scale13** | **Caregiver gave consent** |
| **9** | **Bektaş- Kayhan, K. (9)** | **—** | **18** | **RMS** | **CT, RT** | **Jaw hypoplasia, trismus, hyposalivation, incomplete dentition, dental caries, stunted root, blunt roots, retained tooth** | **Poor oral health, dental developmental anomalies, impaired oral function and aesthetics** | **14** |  |
| **10** | **Bektaş- Kayhan, K.K (10)** | **—** | **13,14,15 years** | **Nasopharyngeal carcinoma** | **CT, RT** | **Xerostomia, dental caries, trismus** | **Difficulty swallowing speaking, sleeping** | **15** |  |
| **11** | **Bendoraitiene,**  **E.A. (11)** | **—** | **11** | **Central nervous system tumors** | **RT, Surgery** | **Dental caries, poor oral hygiene, gingivitis** | **Cosmetic deformity, adaptive eating strategies, poor**  **oral health** | **16** | **Caregiver gave consent** |
| **12** | **Bertoglio, J.C. (12)** | **29** | **4 month to 17 years** | **Hematological malignancies, solid tumors** | **CT** | **Oral mucositis, oral bleeding, ulcers, bacterial and fungal infections** | **Severe oral pain, difficulty**  **eating, drinking, speaking, odynophagia** | **17** |  |
| **13** | **Bostanabad,**  **M. A. (13)** | **—** | **3-14** | **Acute myeloid leukemia, acute lymphoid leukemia, central nervous system tumor, lymphoma, neuroblastoma, Wilms' tumor, connective**  **tissue sarcoma** | **CT** | **Oral mucositis** | **Oral pain** | **Ou18cher pain scale** | **Participants answered the questionnaire in an interview with the assistance of the HCP**  **Participant self-reported the symptoms** |
| **14** | **Bresolin, Carmela R (14)** | **41** | **3-21** | **Leukemia Lymphoma Sarcoma**  **Wilms’ tumor** | **CT**  **RT** | **Dental caries**  **Gingivitis**  **Oral infections**  **Oral mucositis** | **Cancer treatment was found to significantly reduce the oral health-related quality of life (OHRQoL), particularly in patients aged 11–14 years.**  **Children aged 8–10 years without cancer reported worse OHRQoL than their cancer-diagnosed counterparts** | **Early Childhood Oral Health Impact**  **Scale (ECOHIS), the Child Perceptions Questionnaire (CPQ) 11–14‑short version, the CPQ 8–10‑short version, and the short‑form of Oral**  **Health Impact Profile Questionnaire‑14 (OHIP‑14).** | **Children aged 8 years and older completed the questionnaires themselves.** |
| **15** | **Caran, E.M. (15)** | **—** | **12** | **Hemangiopericytoma** | **CT, RT, Surgery** | **Facial asymmetry, trismus, tooth loss, malocclusion, dental developmental anomalies** | **Impaired oral function and aesthetics, social isolation, psychological impact,** |  |  |
| **16** | **Carneiro, T. V. (16)** | **—** | **5-18** | **Type of cancer not mentioned** | **Not Mentiomed** | **Oral mucositis, dental caries** | **Oral pain, toothache, discomfort, feeling sad, difficulty eating and maintaining oral hygiene due to pain, poor QoL** | **Modified Autoquestionnaire Qualité de Vie Enfant Imagé (AUQEI)** | **Caregiver gave consent**  **Participants answered the questionnaire in an interview** |
| **17** | **Carneiro, T. V. (17)** | **34** | **6-15** | **Central nervous system tumors, leukemia, lymphoma,**  **other solid tumors** | **CT, RT, Surgery** | **Dental caries, oral lesions** | **Smiling with embarrassment, toothache** | **Pediatric Quality of Life Inventory (PedsQL™ 3.0 module)** | **Participants above the age of 12 years answered directly**  **Caregivers of participants below the age of answered for them** |
| **18** | **Cauwels, R. G. (18)** | **—** | **9.4 (mean age)** | **Leukemia, lymphoma, neuroblastoma, osteosarcoma, Ewing sarcoma, germ cell tumor** | **CT** | **Oral mucositis** | **Oral pain, difficulty swallowing and speaking, compromised nutrition** | **Visual Analog Scale (VAS) for pain**  **The World Health Organization (WHO) mucositis scale**  **A questionnaire concerning oral functions, nutrition, speech and deglutition, to assess the grade of mucositis** | **Participants answered the questionnaire** |
| **19** | **Chang P.C (19)** | **—** | **7 years-follow till 32 years** | **Rhabdomyosarcoma (RMS)** | **CT**  **RT** | **Tooth and jaw abnormality** | **-Impact psychological states**  **-Severe functional and aesthetic issues affecting mastication and appearance.**  **-As the patient aged, they developed depression due to facial deformities and dental problems** | **NR** | **NR** |
| **20** | **Cheng, K.K.F. (20)** | **43** | **6-18** | **Hematological malignancies, solid tumors** | **CT** | **Oral mucositis** | **Difficulty swallowing, drinking, eating, speaking and sleeping** | **Mouth and Throat Soreness-**  **Related Questions of the Oral Mucositis Daily Questionnaire (OMDQ MTS)**  **Short form of the Chinese version of the State Anxiety Scale for Children (CSAS-C)** | **Participants provided assent and their caregiver gave consent**  **Participants completed the questionnaire with the assistance of caregivers** |
| **21** | **Cheng, K.K.F. (21)** | **37** | **6-18** | **Hematological malignancies, solid tumors** | **CT** | **Oral mucositis, sialorrhea, alterations in saliva consistency** | **Severe oral pain, difficulty speaking, drinking, sleeping, psychological distress** | **Chinese version of the Oral**  **Mucositis Daily Questionnaire (OMDQ)**  **Oral Mucositis- specific Quality of Life Measure (OMQoL)** | **Participants self- reported symptoms and completed the questionnaire with the assistance of caregivers, if needed** |
| **22** | **Chouksey,**  **G.C. (22)** | **—** | **6** | **Acute lymphoid leukemia** | **CT** | **Palatal perforation** | **Difficulty in eating,**  **swallowing, and nasal regurgitation, impaired speech** |  |  |
| **23** | **Cockerill, C. (23)** | **—** | **3-18** | **Salivary gland tumor** | **CT, RT, Surgery** | **Facial twitching, xerostomia, facial paralysis, gustatory sweating (Frey's syndrome), secondary malignancy, jaw hypoplasia, sialorrhea** | **Facial paralysis, numbness and pain, dental developmental anomalies, impaired speech, difficulty eating, impaired oral function, nutritional support, phycological impact (depression), self-conscious about appearance** | **Phone interview and Patients were also contacted by phone for a follow up survey** | **Participants provided consent**  **(206)** |
| **24** | **Czyzewski, K. (24)** | **—** | **median, 10.4 vs**  **13.2 years (study vs control)** | **Lymphoma, neuroblastoma or other solid tumors** | **HSCT** | **Oral mucositis** | **Oral pain, unable to eat and swallow** | **WHO mucositis scale** | **HCP examined the participant** |
| **25** | **de Oliveira, I. S. (25)** | **—** | **9** | **Neuroblastoma** | **CT** | **Mucositis** | **Sever pain, dysphagia, suppressing the ability to eat contributing to malnutrition, severe**  **weight loss and worsening quality of life** | **NR** | **NR** |
| **26** | **Dev, K. (26)** | **—** | **3-18** | **Ewing sarcoma** | **CT, RT, Surgery** | **Secondary malignancy** | **Oral pain** |  |  |
| **27** | **Elad, S. (27)** | **—** | **13-16** | **Burkitt lymphoma, Ewing sarcoma, osteosarcoma,** | **CT** | **Oral mucositis** | **Oral pain** | **WHO mucositis scale**  **The Oral Mucositis Assessment**  **Scale (OMAS)**  **Visual Analog pain scale**  **(patient reporting scale of 0-10)** | **Participant self-reported the symptoms**  **caregivers gave consent** |
| **28** | **Fistikci, Y. (28)** | **—** | **8-18 mean 11.11 ±2.51 y** | **leukemia, lymphoma, Brain tumor, Wilms tumor** | **CT** | **Loss of taste** | **A negative relationship was determined between food cravings and enjoyment of food and altered taste perception.**  **Inconvenience in daily life** | **Children’s Eating Behavior Questionnaire (CEBQ)** | **The questionnaire was administered to the children included in the research** |
| **29** | **Gandhi, K. (29)** | **—** | **2-14** | **lymphoma leukemia, osteosarcoma,**  **stem cell glioma, astrocytoma, carcinoma lung, neuroblastoma, Ewing sarcoma, anaplastic astrocytoma, carcinoma in situ,**  **hepatoblastoma** | **CT** | **Oral mucositis, head and neck lymphadenopathy, cracked lip, ulcers, herpes simplex infection temporomandibular joint pain,**  **oral petechiae, ecchymosis, gingivitis, fungal infection (candidiasis) xerostomia** | **Oral pain, dry mouth, difficulty in swallowing and speaking** | **WHO mucositis scale** | **Caregiver and HCP gave consent** |
| **30** | **Garrocho- Range, J.A. (30)** | **30** | **3-12** | **Acute lymphoid leukemia** | **CT** | **Oral mucositis, cracked lips, coated tongue, angular cheilitis** | **Oral pain** | **WHO mucositis scale** | **Caregiver gave consent** |
| **31** | **Guimarães, J.R. (31)** | **—** | **1-18** | **Acute myeloid leukemia, acute lymphoid leukemia, non-Hodgkin’s lymphoma, Hodgkin’s lymphoma, solid malignant tumors** | **CT** | **Oral mucositis** | **Oral pain** | **Modified Oral Assessment Guide (OAG)** |  |
| **32** | **Hendrawati S. (32)** | **36** | **2-18 years** | **NR** | **CT** | **Mucositis** | **There is a significant relationship between mucositis and QoL of children with cancer**  **Functional disorder: gnawing, swallowing and speaking obstruction.**  **subjective changes: pain, sensitivity and dry feeling** | **PedsQoL Cancer Module 3.0.** | **NR** |
| **33** | **Hernandez, M. (33)** | **—** | **7** | **Acute myeloid leukemia** | **CT, HSCT** | **Enamel defects,**  **microdontia, stunted root, hypodontia, malocclusion** | **Dental**  **developmental anomalies, cosmetic deformity, difficulty chewing, root abnormalities, failure to perform orthodontic treatment due to dental anomalies** |  | **Participants and their caregiver mutually approved the treatment plan** |
| **34** | **Hong, H.C. (34)** | **—** | **19-39** | **CNS tumor, leukemia, lymphoma, solid tumor, other** | **CT, RT, HSCT, Surgery** | **Xerostomia, mouth sores** | **Taste changes, oral pain, difficulty swallowing and sleeping, lack of concentration and energy, feeling sad, depressed, anxious** | **Memorial Symptom Assessment Scale (MSAS)** | **Participants completed the surveys** |
| **35** | **Horri, A. (35)** | **—** | **8** | **Acute myeloid leukemia** | **CT** | **Necrotic fungal infection, facial asymmetry** | **Difficulty in eating and speaking, cosmetic deformity, asymmetrical smile, social isolation, lack of self-confidence, psychosocial impact, missing school** |  |  |
| **36** | **Inati, A. (36)** | **—** | **5** | **Acute lymphoid leukemia** | **CT** | **Oral mucositis, necrotic and hemorrhagic lesions in mouth, dental caries** | **Unable to eat, drink and sleep** |  |  |
| **37** | **Ip, W.Y. (37)** | **44** | **6-18** | **Haematological malignancies, solid tumors** | **CT** | **Oral mucositis, sialorrhea** | **Oral and throat pain, difficulty swallowing, eating, sleeping and speaking** | **Chinese**  **version of the State Anxiety Scale for Children (CSAS-C)**  **Oropharyngeal Mucositis Quality of Life Scale (OMQoL)** | **Caregiver gave consent** |
| **38** | **Kamasaki, Y. (38)** | **—** | **6-18** | **Acute lymphoid leukemia** | **CT, HSCT** | **Cellulitis, trismus** | **Poor oral health, severe oral pain, impaired aesthetic due to healing scar** |  |  |
| **39** | **Khurana, H. (39)** | **46** | **6-15** | **Acute lymphoid leukemia, acute myeloid leukemia, non-Hodgkin’s lymphoma** | **CT** | **Oral mucositis** | **Odynophagia, compromised nutrition, distress** | **WHO mucositis scale**  **OMAS**  **Children’s International Mucositis**  **Evaluation Scale (ChIMES)** | **Participant self-reported the symptoms caregiver gave consent** |
| **40** | **Kilic M. (40)** | **47** | **87.12 ± 35.04 month**  **92%under 6 years** | **Acute lymphocytic leukemia**  **Acute myelocytic leukemia** | **NR** | **NR** | **Children in the cancer group had significantly more loss of function (eating, drinking, speaking, missed school) than those in the control group** | **Early Childhood Oral Health Impact Scale (ECOHIS-­T)** | **Quality of life was evaluated based on the report of parents.** |
| **41** | **King, E. (41)** | **—** | **15, 18, 20** | **Neuroblastoma and head and neck RMS** | **CT, RT** | **Teeth agenesis, hypoplasia, microdontia, root malformation, delayed eruption, malocclusions, dental caries, periodontitis** | **Aesthetic problem, eating problems** |  |  |
| **42** | **Korfage, A. (42)** | **—** | **24, 25** | **Nasopharyngeal carcinoma, RMS** | **CT, RT, Surgery** | **Microdontia, root malformation, mid- face hypoplasia, trismus, jaw hypoplasia, hypodontia, microstomia, malformed teeth** | **Impaired oral function such as speaking problems and aesthetic impairment,** |  |  |
| **43** | **Kostak, M. A. (43)** | **45** | **8-18** | **Acute lymphoid leukemia** | **CT** | **Oral mucositis** | **Oral pain** | **WHO mucositis scale**  **ChIMES** | **Participant self-reported the symptoms** |
| **44** | **Lauritano, D. (44)** | **—** | **7-16** | **Acute lymphoid leukemia** | **HSCT** | **Oral mucositis** | **Oral pain,** | **WHO mucositis scale** | **Caregiver gave consent** |
| **45** | **Leiser, D. (45)** | **—** | **3-16** | **RMS** | **CT,**  **pencil**  **beam scanning (PBS), proton therapy (PT)** | **Impaired dental growth, facial hypoplasia** | **Cosmetic deformity, dental developmental anomalies** | **PedQoL Questionnaire:**  **The proxy-rating version (PedQoL proxy)**  **self-rating version for children older**  **than 4 years (PedQoL self)** | **Caregivers completed the questionnaire for the participants** |
| **46** | **Liu, Y. (46)** | **—** |  | **Acute myeloid leukemia** | **CT, HSCT** | **chronic GVHD, secondary malignancy** | **Oral pain** |  |  |
| **47** | **Loves, R. (47)** | **—** | **4-18** | **Central nervous system tumor, leukaemia, lymphoma, solid tumour, other** | **CT, RT, Surgery, HSCT** | **Dysgeusia** | **Loss of appetite, unable to enjoy food, adaptive eating strategies, increased hydration, frequently brushing teeth due to bad taste** | **Structured interview**  **Symptom Screening in Pediatrics Tool (SSPedi) (age 8–18)**  **mini-SSPedi (age 4–7)** | **Participants provided assent and their caregivers gave consent Participant self-reported the symptoms** |
| **48** | **Loves, R. (47, 48)** | **—** | **4-18** | **Leukemia, lymphoma, solid tumor, brain tumor, other types** | **CT, RT, HSCT, Surgery** | **Oral mucositis**  **, fungal infection (thrush), dysgeusia** | **Taste changes,** | **Single semi-structured interview**  **Symptom Screening in Pediatrics Tool (SSPedi) (age 8–18)**  **mini-SSPedi (age 4–7)** | **Participants and their caregiver gave consent**  **Participant self-reported the symptoms** |
| **49** | **Lucchese, A. (49)** | **—** | **7-16** | **Acute lymphoid leukemia** | **CT** | **Oral mucositis** | **Oral pain, unable to**  **eat, drink, swallow, or speak** | **WHO mucositis scale OMDQ** | **Caregiver gave consent**  **Participants answered the questionnaire** |
| **50** | **Lupi, S.M. (50)** | **—** | **25** | **Acute myeloid leukemia** | **CT,RT** | **Hypodontia, mobile teeth, root hypoplasia and malformation, dental caries** | **Toothache, dental developmental anomalies** |  |  |
| **51** | **Manji, A. (51)** | **42** | **12-18** | **Acute myeloid leukemia, relapsed acute lymphoid leukemia, advanced lymphoma, solid tumors, brain tumor** | **CT** | **Oral mucositis** | **Oral pain, difficulty swallowing, drinking, and talking** | **OMDQ**  **WHO mucositis scale**  **VAS for pain**  **Functional Assessment of Cancer**  **Therapy Esophageal Cancer Sub-scale (FACT-ECS).** | **Participants self-reported the symptoms** |
| **52** | **Marangoni-Lopes, L. (52)** | **51** | **6-16** | **Hodgkin’s lymphoma** | **CT,RT** | **Xerostomia** | **Oral pain, burning sensation, difficulty swallowing, speaking, eating, trismus,**  **opening mouth, poor QoL** | **Quality of Life – Head and Neck module 35 questionnaire (QLQ- HN 35)** | **Caregiver gave consent**  **Participants completed the questionnaire along with their caregiver** |
| **53** | **Martin, P. (53)** | **—** | **18** | **Bilateral retinoblastoma** | **RT** | **Hypodontia, microdontia, jaw hypoplasia, malocclusion** | **Cosmetic deformity, psychosocial impact, impaired oral function, discomfort, dental developmental anomalies** |  | **Participants and their caregiver were motivated to obtain dental treatment** |
| **54** | **Michalak, I. (54)** | **—** | **0.1 month-17.7years** | **RMS, Burkitt’s lymphoma** | **CT, RT, Surgery** | **Root agenesis, missing tooth bud, delayed root formation, jaw hypoplasia, facial hypoplasia, demineralised enamel, resorption of jawbone, Dental and craniofacial developmental anomalies** | **difficulty biting and chewing, impaired speech, cosmetic deformity, poor oral health** |  |  |
| **55** | **Miranda-Silva,**  **W. (55)** | **—** | **0-17** | **Acute myeloid leukemia, acute lymphoid leukemia, juvenile myeloid leukemia, other cancer** | **CT, TBI, HSCT** | **Oral mucositis, sialorrhea, xerostomia** | **Odynophagia, nutritional deficiency** |  |  |
| **56** | **Najafi, S.H. (56)** | **—** | **26 years at the time of study** | **Hodgkin’s lymphoma** | **CT, RT** | **Root resorption, stunted roots, mobile teeth, gingivitis, root malformation** | **Difficulty eating and maintaining oral hygiene, poor oral health** |  |  |
| **57** | **Nielsen, B. N. (57)** | **—** | **2-17** | **Type of cancer not mentioned** | **CT** | **Oral mucositis** | **Oral pain, Odynophagia and pain while brushing teeth** | **VAS modified with six faces for pain**  **Face Leg Activity Cry Consolability (FLACC) scale**  **WHO mucositis scale** | **Participants self-reported the symptoms** |
| **58** | **Noirrit- Esclassan, E. (58)** | **—** | **3-18** | **Leukemia, CNS tumor, lymphoma, bone tumor, non-bone sarcoma** | **CT, RT** | **Oral mucositis** | **Oral pain, unable to eat** | **WHO mucositis scale**  **HEDEN mucositis scale**  **VAS for pain** | **Participants and their caregiver gave consent** |
| **59** | **Okello DA (59)** | **48** | **8-12** | **Acute Lymphoblastic Leukemia** | **CT**  **RT**  **Surgery** | **Oral mucositis and dental caries** | **Oral complications affecting children's ability to eat, talk, chew, and swallow, leading to discomfort and a decreased quality of life** | **-Child - Oral Impacts on Daily Performance**  **Questionnaire**  **-Child Perception Questionnaire**  **Child (CPQ)** | **The questionnaire was administered to the children included in the research** |
| **60** | **Ozdere, E. (60)** | **31** | **13** | **Nasopharyngeal carcinoma** | **CT,RT** | **Trismus, dental caries** | **Jaw pain** |  |  |
| **61** | **Padmanabhan,**  **M.Y. (61)** | **—** | **15** | **Maxillofacial Burkitt lymphoma** | **CT** | **Oral mucositis**  **, dental caries, xerostomia** | **Difficulty chewing and swallowing, dry mouth** |  |  |
| **62** | **Pels, E. (62)** | **—** | **2-18** | **Acute lymphoid leukemia** | **CT** | **Oral mucositis**  **, fungal infection** | **Oral pain** |  |  |
| **63** | **Popescu, B. (63)** | **—** | **6-13** | **Well-differentiated squamous**  **cell carcinoma and**  **Ewing sarcoma** | **CT, RT,Surgery** | **Tongue resection** | **Impaired oral function and aesthetic, healing scars, altered voice, impaired speech, social isolation, difficulty speaking** |  |  |
| **64** | **Pourdeghatkar,**  **F. (64)** | **—** | **6-15** | **Acute myeloid leukemia** | **CT, RT, HSCT, Surgery** | **Oral mucositis** | **Oral pain, adaptive eating strategies** | **WHO mucositis scale** | **Caregiver gave consent**  **HCP examined the participant** |
| **65** | **Ridho, F. (65)** | **—** | **9** | **Leukemia** | **CT** | **-Angioedema on the lips**  **-Sero sanguinolent crust**  **-White plaques** | **Negatively impact quality of life swelling on the lips, difficulty while eating, swallowing, and even opening the mouth** | **NR** | **NR** |
| **66** | **Rimulo, A.L (66)** | **—** | **5** | **Acute myeloid leukemia** | **CT** | **Oral mucositis** | **Oral pain** | **WHO mucositis scale**  **VAS for pain** | **Participants self-reported the symptoms** |
| **67** | **Saha, A. (67)** | **54** | **11.4-22.5** | **Central nervous system tumors** | **CT, RT, HSCT, Surgery** | **Microdontia, hypodontia, root malformation, dental caries, gingivitis** | **Dental developmental anomalies, impaired speech, altered taste, psychosocial impairment** | **Behavior Assessment System for Children, 2nd**  **Edition (BASC-2)**  **Children’s Health Questionnaire, Parent**  **Form (CHQ-PF50)**  **Childhood Cancer Survivor Study (CCSS) questionnaire** | **Participants (minors) provided assent; caregiver gave consent and completed the questionnaire**  **Adult survivors provided consent and completed the**  **questionnaire** |
| **68** | **Scarpelli, A.C. (68)** | **—** | **14** | **Nasopharyngeal carcinoma** | **CT, RT** | **Discolored teeth, dental caries, xerostomia, dry lips** | **Toothache, psychosocial impact, impaired aesthetics** |  | **Participant and the caregiver both reported the medical history and examination** |
| **69** | **Shahriari, M. (69)** | **—** | **0-18** | **Acute lymphoid leukemia** | **CT, HSCT** | **Mucosal hypertrophy, leukemia cutis (relapse of leukemia due to infiltration in oral mucosa and skin)** | **Burning sensation, oral pain** |  |  |
| **70** | **Shum, M. (70)** | **39** | **14-16** | **RMS, central nervous system tumor, leukemia, lymphoma, kidney tumor, retinoblastoma, other**  **sarcoma’s and cancer** | **CT, RT, HSCT, Surgery** | **Tooth agenesis, microdontia, root abnormalities** | **Functional, psychological, social disability** | **Holtta’s Defect Index (HDI)**  **Oral Health Impact Profile-14 (OHIP-14)** | **Participants answered the survey Participants above 16**  **years of age solely gave consent**  **Both participants (below 16) and their caregivers gave consent** |
| **71** | **Soares, A. D.**  **S. (71)** | **10** | **4-12** | **Leukemia** | **CT** | **Oral mucositis** | **Oral pain** | **WHO mucositis scale**  **Wong-Baker visual analog scale for pain** | **Evaluator examined the participant** |
| **72** | **Stolze J. (72)** | **40** | **median age at study enrollment was 32.3 years (range 16.8–59.5)** | **NR** | **CT**  **RT**  **HSCT** | **Oral health problems (oral blisters/aphthae and ‘bad odor/halitosis)**  **Dental problems (cavities, gingival problems, and sensitive exposed root surfaces)** | **Perceived OHRQoL overall was relatively good**  **OHRQoL was more associated with the number of oral complications and younger age at study enrollment and a shorter time since childhood cancer diagnosis showed higher number of oral health problems** | **Oral Health Impact Profile-14 (OHIP-14)** | **Self-reported by the child** |
| **73** | **Tummawanit,**  **S. (73)** | **32** | **16** | **Retinoblastoma** | **CT, RT, Surgery** | **Jaw hypoplasia, microdontia, hypodontia, skeletal malocclusion** | **Dental and orofacial developmental anomalies, cosmetic deformity,**  **distress** |  |  |
| **74** | **Vagliano, L. (74)** | **—** | **0-18** | **Central nervous system tumors** | **HSCT** | **Oral mucositis** | **Oral pain, compromised nutrition** | **WHO mucositis scale** | **Healthcare provider (HCP’s) examined the participant** |
| **75** | **Van den Brink, M. V. (75)** | **33** | **6-18** | **Acute myeloid leukemia, acute lymphoid leukemia, RMS**  **lymphoma, medulloblastoma, bone tumor** | **CT** | **Dysgeusia, hypogeusia** | **Taste changes, appetite loss, eating disorders** | **Behavioral Pediatrics Feeding Assessment Scale (BPFAS)**  **Likert scale (1 “very bad” to 5 “very good”) for assessment for smell, taste and appetite** | **Consent was obtained from Caregiver and participants ≥ 12 years**  **Participants assessed and reported the symptoms**  **Caregivers reported the BPFAS** |
| **76** | **Wang, Y. (76)** | **—** | **Under18** | **Acute lymphoid leukemia** | **CT** | **Dental caries, gingivitis, xerostomia** | **Poor oral health, loss of appetite, eating problems** |  | **Researchers examined the participant** |
| **77** | **White, M.C. (77)** | **—** | **3-14** | **Acute lymphoid leukemia, acute myeloid leukemia, non-Hodgkin’s lymphoma, primitive neuroectodermal tumor, other** | **CT, HSCT** | **Oral mucositis** | **Oral pain** | **NCI-CTC scales for OM**  **Wong and Baker Faces with a 0–10 numerical rating scale**  **VAS for pain FLACC scale** | **Participant self-reported the symptoms; if the participant could not report the HCP used an observational tool (FLACC)** |
| **78** | **Williams, P.D. (78)** | **35** | **5-17** | **Acute myeloid leukemia, solid tumors, nervous system tumors,**  **Other** | **CT, RT** | **Oropharyngeal symptoms, dysphagia** | **Jaw pain, difficulty swallowing, soreness in**  **mouth and throat** | **Therapy-Related Symptom Checklist Children (TRSC-C)**  **PedsQL™ 3.0 module** | **Participants self- reported symptoms; the caregivers and HCP could assist the participants to complete the checklist, if needed** |
| **79** | **Yavuz, A. (79)** | **—** | **17** | **Nasopharyngeal carcinoma** | **CT,RT** | **Xerostomia, dental caries, thermosenstivity, gingivitis, trismus, altered teeth development** | **Difficulty chewing and speaking, dry mouth psychosocial distress, cosmetic deformity** |  | **Caregiver gave consent** |

NR (Not Referred): Refers to information not referred to in the study

1. Agholme MB, Dahlloef G, Toerlen JK, Majorana A, Brennan MT, von Bueltzingsloewen I, et al. Incidence, severity, and temporal development of oral complications in pediatric allogeneic hematopoietic stem cell transplant patients - a multicenter study. Supportive Care in Cancer. 2023;31(12).

2. Alonso-Prieto M, Miro J, Torres-Luna R, de Sabando DPL, Reinoso-Barbero F. The association between pain relief using video games and an increase in vagal tone in children with cancer: analytic observational study with a quasi-experimental pre/posttest methodology. Journal of Medical Internet Research. 2020;22(3):8.

3. Amadori F, Bardellini E, Conti G, Pedrini N, Schumacher RF, Majorana A. Low-level laser therapy for treatment of chemotherapy-induced oral mucositis in childhood: a randomized double-blind controlled study. Lasers in medical science. 2016;31:1231-6.

4. Arpaci T, Toruner EK, Altay N. Assessment of nutritional problems in pediatric patients with cancer and the information needs of their parents: a parental perspective. Asia-Pacific Journal of Oncology Nursing. 2018;5(2):231-6.

5. Attinà Giorgio G. Management of oral mucositis in children with malignant solid tumors. Frontiers in Oncology,. 2021;11.

6. Bardellini E, Amadori F, Majorana A. Oral hygiene grade and quality of life in children with chemotherapy-related oral mucositis: a randomized study on the impact of a fluoride toothpaste with salivary enzymes, essential oils, proteins and colostrum extract versus a fluoride toothpaste without menthol. International Journal of Dental Hygiene. 2016;14(4):314-9.

7. Bardellini E, Amadori F, Schumacher RF, D'Ippolito C, Porta F, Majorana A. Efficacy of a solution composed by verbascoside, polyvinylpyrrolidone (PVP) and sodium hyaluronate in the treatment of chemotherapy-induced oral mucositis in children with acute lymphoblastic leukemia. Journal of Pediatric Hematology and Oncology. 2016;38(7):559-62.

8. Bardellini E, Amadori F, Schumacher RF, Foresti I, Majorana A. A new emerging oral infection: raoultella planticola in a boy with haematological malignancy. European Archives of Paediatric Dentistry. 2017;18(3):215-8.

9. Bektaş-Kayhan K, Karagöz G, Bayrak Ö, Kürklü E, Özbek CD, Ak G, et al. Implant-assisted dental rehabilitation of a patient with maxillary rhabdomyosarcoma. Journal of Craniofacial Surgery. 2012;23(5):e384-6.

10. Bektas-Kayhan KK. Long-term maxillofacial effects of radiotherapy in young nasopharyngeal carcinoma patients: report of 3 cases. The Journal of Clinical Pediatric Dentistry. 2013;37(4):407-10.

11. Bendoraitiene Egle Aida EA. Peculiarities of dental treatment among paediatric oncological patients: a case report. Journal of Oral Maxillofacial Research. 2020;11(3).

12. Bertoglio JC, Folatre I, Bombardelli E, Riva A, Morazzoni P, Ronchi M, et al. Management of gastrointestinal mucositis due to cancer therapies in pediatric patients: results of a case series with SAMITAL(®). Future Oncology. 2012;8(11):1481-6.

13. Bostanabad MA, Hiradfar A, Mohammadpoorasl A, Javadzadeh Y, Khalvati B, Alvandnezhad T. The effect of mucoadhesive gel containing satureja hortensis extract 1% on severity of chemotherapy-induced mucositis Pain in children: a randomized clinical trial. Int J Pediatr-Masshad. 2018;6(5):7605-14.

14. Bresolin CR, Palma LF, Serrano RV, Toline C, Tuchtenhagen S, Ardenghi TM, et al. Oral health-related quality of life in Brazilian children, adolescents, and young adults undergoing cancer treatment. National Journal of Maxillofacial Surgery. 2023;14(3):383-7.

15. Caran EMM, Barone TR, Barone JR, Lopes NNF, Alves MTS, França CM. Facial reconstruction surgery 10 years after treatment for hemangiopericytoma: planning considerations and clinical outcomes. J Cosmet Laser Ther. 2014;16(4):201-4.

16. Carneiro TV, de Lucena RB, Ribeiro ILA, Agripino GG, Valenca AMG, da Rosa MRD. Quality of life of paediatric oncology patients. Pesquisa Brasileira Em Odontopediatria E Clinica Integrada. 2016;16(1):457-67.

17. Carneiro TV, Ribeiro ILA, Alves CV, Bonan PRF, Lima Neto EA, Valenca AMG. Factors associated with health-related quality of life among children with cancer from the standpoint of patients and caregivers. Journal of Public Health (Germany). 2017;25(4):371-7.

18. Cauwels RG, Martens LC. Low level laser therapy in oral mucositis: a pilot study. European Archives of Paediatric Dentistry. 2011;12(2):118-23.

19. Chang PC, Lin SY, Chang P-C, Lin S-Y. A Long-Term Follow-Up of Dental and Craniofacial Disturbances after Cancer Therapy in a Pediatric Rhabdomyosarcoma Patient: Case Report. INTERNATIONAL JOURNAL OF ENVIRONMENTAL RESEARCH AND PUBLIC HEALTH. 2021;18(22).

20. Cheng KKF, Lee V, Li CH, Goggins W, Thompson DR, Yuen HL, et al. Incidence and risk factors of oral mucositis in paediatric and adolescent patients undergoing chemotherapy. Oral Oncology. 2011;47(3):153-62.

21. Cheng KKF, Lee V, Li RCH, Yuen HL, Epstein JB. Oral mucositis in paediatric patients undergoing chemotherapy: impact on oral functional status and quality of life. Supportive Care in Cancer. 2011;19(2):S246.

22. Chouksey Gunjan CG. Prosthetic management of hard palate perforation in a child with acute lymphoblastic leukemia. Indian Journal of Medical and Paediatric Oncology : Official Journal of Indian Society of Medical and Paediatric Oncology. 2017;38(2):220-2.

23. Cockerill CC, Gross BC, Contag S, Rein S, Moore EJ, Olsen KD, et al. Pediatric malignant salivary gland tumors: 60 year follow up. International Journal of Pediatric Otorhinolaryngology. 2016;88:1-6.

24. Czyzewski K, Debski R, Krenska A, Wysocki M, Styczynski J. Palifermin in children undergoing autologous stem cell transplantation: a matched-pair analysis. Anticancer Research. 2014;34(12):7379-82.

25. de Oliveira IS, de Deus LP, Esteves TC, das Gracas Afonso Miranda Chaves M, Fabri JC, Fabri GMC. Therapeutic challenges of oral mucositis in pediatric oncology. Medicina. 2023;56(2):e-184330.

26. Dev K. Solitary gingival metastases in fibular ewing's sarcoma. Indian Journal of Surgical Oncology. 2016;7 (3):290.

27. Elad Sharon S. Topical curcumin for the prevention of oral mucositis in pediatric patients: case series. Alternative Therapies in Health and Medicine. 2013;19(3):21-4.

28. Fistikci Y, Kocamaz EB. Eating behavior, nutritional status, and taste perception alteration in children with cancer. Journal of Pediatric Hematology/Oncology. 2024;46(2):88 EP - 95.

29. Gandhi K, Datta G, Ahuja S, Saxena T, A GD. Prevalence of oral complications occurring in a population of pediatric cancer patients receiving chemotherapy. International Journal of Clinical Pediatric Dentistry. 2017;10(2):166-71.

30. Garrocho-Rangel JA, Herrera-Moncada M, Márquez-Preciado R, Tejeda-Nava F, Ortiz-Zamudio JJ, Pozos-Guillén A. Oral mucositis in paediatric acute lymphoblastic leukemia patients receiving methotrexate-based chemotherapy: case series. European Journal of Paediatric Dentistry. 2018;19(3):239-42.

31. Guimarães JRJ. The incidence of severe oral mucositis and its occurrence sites in pediatric oncologic patients. Medicina Oral Patologia Oral y Cirugia Bucal. 2021;26(3):299.

32. Hendrawati S, Nurhidayah I, Mediani HS, Mardhiyah A, Maryam NNA. Mucositis effect on quality of life of hospitalized children with cancer who received chemotherapy. Jurnal Keperawatan Padjadjaran. 2019;7(1):29-37.

33. Hernandez Magali M. Long-term adverse effects of acute myeloid leukemia treatment on odontogenesis in a child. International Journal of Clinical Pediatric Dentistry. 2019;12(3):243-6.

34. Hong HC, Kim YM, Min A. Symptom clusters in childhood cancer survivors in Korea: a latent class analysis. European Journal of Cancer Care. 2020;29(6):14.

35. Horri A, Khademi M, Faryabi J, Shojaeepour R. Aspergillosis in a child with acute myeloid leukemia: complications, and treatment. Journal of Dentistry (Shiraz). 2018;19(4):320-4.

36. Inati Adlette A. A rare aggravation of severe mucositis post chemotherapy in a child with acute lymphoblastic leukemia. F1000Research. 2013;2.

37. Ip WY, Epstein JB, Lee V, Yuen HL, Li R, Thompson DR, et al. Oral mucositis in paediatric patients after chemotherapy for cancer. Hong Kong Med J. 2014;20(6):4-8.

38. Kamasaki Y, Satoh K, Nishiguchi M, Hoshino T, Fujiwara T. Acute oral complications in a pediatric patient with acute lymphoid leukemia. Pediatrics International. 2016;58(6):484-7.

39. Khurana H, Pandey RK, Saksena AK, Kumar A. An evaluation of Vitamin E and Pycnogenol in children suffering from oral mucositis during cancer chemotherapy. Oral Diseases. 2013;19(5):456-64.

40. Kilic M, Yilmaz SG, Kockopru ZM, Kilic M, Yilmaz SG, Kockopru ZM. Evaluation of oral health-related quality of life in children with acute lymphocytic leukemia/acute myelocytic leukemia: A cross-sectional study. Oral Diseases. 2024;30(4):2663-9.

41. King E. Oral sequelae and rehabilitation considerations for survivors of childhood cancer. British Dental Journal. 2019;226(5):323-9.

42. Korfage A, Stellingsma K, Jansma J, Vissink A, Raghoebar GM. Oral rehabilitation with implant-based prostheses of two adult patients treated for childhood rhabdomyosarcoma. Supportive Care in Cancer. 2011;19(9):1477-80.

43. Kostak MA, Semerci R, Eren T, Kocaaslan EN, Yildiz F. Effects of oral health care education on the severity of oral mucositis in pediatric oncology patients. Turkish Journal of Oncology. 2020;35(4):422-9.

44. Lauritano D, Petruzzi M, Di Stasio D, Lucchese A. Clinical effectiveness of palifermin in prevention and treatment of oral mucositis in children with acute lymphoblastic leukaemia: a case-control study. International Journal of Oral Science. 2014;6(1):27-30.

45. Leiser D, Calaminus G, Malyapa R, Bojaxhiu B, Albertini F, Kliebsch U, et al. Tumour control and quality of life in children with rhabdomyosarcoma treated with pencil beam scanning proton therapy. Radiotherapy and Oncology. 2016;120(1):163-8.

46. Liu Yanbin Y. In situ buccal carcinoma in a teenager after hematopoietic stem cell transplantation: a case report. Medicine. 2020;99(43).

47. Loves R, Green G, Joseph-Frederick Z, Palmert S, Plenert E, Schechter T, et al. Describing taste changes and their potential impacts on paediatric patients receiving cancer treatments. BMJ support. 2021;28:28.

48. Loves R, Plenert E, Tomlinson V, Palmert S, Green G, Schechter T, et al. Changes in taste among pediatric patients with cancer and hematopoietic stem cell transplantation recipients. Quality of Life Research. 2019;28(11):2941-9.

49. Lucchese Alessandra A. Efficacy and effects of palifermin for the treatment of oral mucositis in patients affected by acute lymphoblastic leukemia. Leukemia and Lymphoma. 2016;57(4):820-7.

50. Lupi Saturnino Marco SM. Long-term effects of acute myeloid leukemia treatment on the oral system in a pediatric patient. Open Dentistry Journal. 2018;12:230-7.

51. Manji A, Tomlinson D, Ethier MC, Gassas A, Maloney AM, Sung L. Psychometric properties of the oral mucositis daily questionnaire for child self-report and importance of mucositis in children treated with chemotherapy. Support Care Cancer. 2012;20(6):1251-8.

52. Marangoni-Lopes L, Rodrigues LP, Mendonça RH, Nobre-Dos Santos M. Radiotherapy changes salivary properties and impacts quality of life of children with hodgkin disease. Archives of Oral Biology. 2016;72:99-105.

53. Martin P, Muller E, Paulus C. Alteration of facial growth after radiotherapy: orthodontic, surgical and prosthetic rehabilitation. Journal of Stomatology, Oral and Maxillofacial Surgery. 2019;120(4):369-72.

54. Michalak Izabela I. Radiological imaging and orthodontic treatment in the case of growing patients after oncological treatment: case reports. Dental and Medical Problems. 2019;56(2):209-15.

55. Miranda-Silva W, da Fonseca FP, Gomes AA, Mafra ABB, Rocha V, Fregnani ER. Oral mucositis in paediatric cancer patients undergoing allogeneic hematopoietic stem cell transplantation preventively treated with professional dental care and photobiomodulation: Incidence and risk factors. International Journal of Paediatric Dentistry. 2021;32(2):251-63.

56. Najafi Sh S. The long-term effects of chemo radiotherapy on oral health and dental development in childhood cancer. Journal of Dentistry of Tehran University of Medical Sciences. 2011;8(1):39-43.

57. Nielsen BN, Aagaard G, Henneberg SW, Schmiegelow K, Hansen SH, Rømsing J. Topical morphine for oral mucositis in children: dose finding and absorption. Journal of Pain and Symptom Management. 2012;44(1):117-23.

58. Noirrit-Esclassan E, Valera MC, Vignes E, Munzer C, Bonal S, Daries M, et al. Photobiomodulation with a combination of two wavelengths in the treatment of oral mucositis in children: the PEDIALASE feasibility study. Archives de Pédiatrie. 2019;26(5):268-74.

59. Okello DA. Dental caries, oral mucositis and oral-health related-quality-of-life in children undergoing cancer therapy at Kenyatta National Hospital: University of Nairobi Research Archieve; 2022.

60. Ozdere E, Ozel GS, Aykent F. Management of restricted mouth opening caused by radiation: a clinical report. Journal of Prosthetic Dentistry. 2016;115(3):263-6.

61. Padmanabhan MY, Pandey RK, Kumar A, Radhakrishnan A. Dental management of a pediatric patient with burkitt lymphoma: a case report. Special Care in Dentistry. 2012;32(3):118-23.

62. Pels Elzbieta E. Oral mucositis in children suffering from acute lymphoblastic leukaemia. Contemporary Oncology. 2012;16(1):12-5.

63. Popescu B, Oancea ALA, Arjoca EM, Androne RG, Mitran DM, Curca C, et al. Malignant tumors of oral cavity in children: cases presentation. Archives of the Balkan Medical Union. 2020;55(1):174-81.

64. Pourdeghatkar F, Motaghi M, Darbandi B, BagherSalimi A. The effect of chamomile mouthwash on the prevention of oral mucositis caused by chemotherapy in children with acute lymphoblastic leukemia. Iran J Pediatr Hematol Oncol. 2017;7(2):76-81.

65. Ridho F, Fitriasari N, Zakiawati D. Efficacy of chlorine dioxide on oral lesions in acute myeloblastic leukemia patients undergoing chemotherapy. International Journal of Applied Pharmaceutics,. 2023;15:52 EP - 7.

66. Rimulo AL, Ferreira MC, Abreu MH, Aguirre-Neto JC, Paiva SM. Chemotherapy-induced oral mucositis in a patient with acute lymphoblastic leukaemia. European Archives of Paediatric Dentistry. 2011;12(2):124-7.

67. Saha A, Salley CG, Saigal P, Rolnitzky L, Goldberg J, Scott S, et al. Late effects in survivors of childhood CNS tumors treated on head start I and II protocols. Pediatric Blood and Cancer. 2014;61(9):1644-52; quiz 53-72.

68. Scarpelli AC, Bendo CB, Novaes-Júnior JB, Barreiros ID, Paiva SM. Aesthetic management of tooth discolouration: Conservative treatment for a patient with undifferentiated nasopharyngeal carcinoma. Rev Odonto Cienc. 2011;26(1):84-7.

69. Shahriari M, Fathpour G, Saleh F. Mucocutaneous relapse as an unusual presentation of T-lineage acute lymphoblastic leukemia. Middle East Journal of Cancer. 2016;7(1):63-6.

70. Shum Matthew M. Associations between childhood cancer treatment and tooth agenesis. The New Zealand Medical Journal. 2020;133(1523):41-54.

71. Soares ADS, Wanzeler AMV, Cavalcante GHS, Barros EMDS, Carneiro RDCM, Tuji FM. Therapeutic effects of andiroba (carapa guianensis aubl) oil, compared to low power laser, on oral mucositis in children underwent chemotherapy: a clinical study. Journal of Ethnopharmacology. 2021;264 (no pagination).

72. Stolze J, Raber-Durlacher JE, Loonen JJ, Teepen JC, Ronckers CM, Tissing WJE, et al. Self-reported outcomes on oral health and oral health-related quality of life in long-term childhood cancer survivors-A DCCSS-LATER 2 Study. Supportive Care in Cancer. 2023;31(6):344.

73. Tummawanit S, Shrestha B, Thaworanunta S, Srithavaj T. Late effects of orbital enucleation and radiation on maxillofacial prosthetic rehabilitation: a clinical report. Journal of Prosthetic Dentistry. 2013;109(5):291-5.

74. Vagliano L, Feraut C, Gobetto G, Trunfio A, Errico A, Campani V, et al. Incidence and severity of oral mucositis in patients undergoing haematopoietic SCT--results of a multicentre study. Bone Marrow Transplant. 2011;46(5):727-32.

75. van den Brink M, Ter Hedde MM, van den Heuvel E, Tissing WJE, Havermans RC. The impact of changes in taste, smell, and eating behavior in children with cancer undergoing chemotherapy: A qualitative study. Frontiers in Nutrition. 2022;9:984101.

76. Wang Y, Zeng X, Yang X, Que J, Du Q, Zhang Q, et al. Oral health, caries risk orofiles, and oral microbiome of pediatric patients with leukemia submitted to chemotherapy. BioMed Research International. 2021;2021 (no pagination).

77. White MC, Hommers C, Parry S, Stoddart PA. Pain management in 100 episodes of severe mucositis in children. Pediatric Anesthesia. 2011;21(4):411-6.

78. Williams PD, Williams AR, Kelly KP, Dobos C, Gieseking A, Connor R, et al. A symptom checklist for children with cancer: the therapy-related symptom checklist-children. Cancer Nurs. 2012;35(2):89-98.

79. Yavuz Atacan A. Multidisciplinary treatment approach in a patient with history of nasopharyngeal carcinoma. Case Reports in Dentistry. 2014;2014.
