# Supplementary material for: The impact of cancer therapy-related oral complications on the quality of life and well-being of childhood cancer survivors: A scoping review
Source: PLoS One. 2026 Jun 23;21(6):e0352194. doi: 10.1371/journal.pone.0352194 (PMC13289919; doi:10.1371/journal.pone.0352194)
Supplement: Attachment — Submitted filename: Response for Reviewers.pdf [file pone.0352194.s006.pdf]

Response Letter

Feb 2, 2026

Giang Truong Vu,

Academic Editor

PLOS ONE

Subject: Submission of revised paper (PONE-D-25-18997)

Dear Academic Editor

Thank you for your email enclosing the reviewers' comments. We have carefully considered all comments and revised the manuscript accordingly. Our detailed responses are provided below in a point-by-point format.

Sincerely,

Nona Attaran

Corresponding author

Comment	Response	Line# in tracked file
**Reviewer 1**		
Abstract: Include protocol registration	The protocol has been included in the abstract.	Line 36-37
Introduction: Consider including the number of children living with cancer (preferably w/in the geographic area of the included articles), and the number of adult survivors of childhood cancers	We have included the estimated number of new children diagnosed with cancer in a five-year rolling period according to the latest update of the Public Health Agency of Canada on cancer in children in Canada. Note: we could not locate a reference for adult survivors.	Line 55-57
Introduction: Line 49: Double-check whether statement "mortality rate continues to decrease" requires some nuance- I suspect this is the case for certain cancers in certain countries (i.e. leukemia in the high-resource countries) but not necessarily for other cancer types and in all resource settings	Thank you for highlighting this important nuance. We agree that childhood cancer mortality remains disproportionately high in low-income countries. Our original statement was intended to reflect the overall global trend, which indicates a decrease in childhood cancer mortality over the past decade. In response to this comment, we have revised the paragraph to explicitly acknowledge this disparity and clarify the distinction between global trends and regional rates.	Line 57-60
Introduction: Would be helpful to provide very specific ways in which this update differs from the 2012 review	We are unclear how to better outline this section. As we mentioned already, this review adds three new dimensions to the 2012 review, as follows:<br><br>1. We have added whether (and how) there has been increased attention to OHRQoL.<br>2. We expanded the scope of the study to include adult survivors of childhood cancer in order to capture the long-term impact of cancer treatment complications on quality of life and well-being.<br>3. We focused on if and how research approaches increasingly acknowledge and incorporate the unique insights, experiences, and perspectives of children surviving cancer on their quality of life and well-being.<br><br>Please advise if this is insufficient.	Line 85-116
Introduction: May want to elaborate more on the types of cancer therapies as how each may impact oral health and subsequently quality of life (for example, children treated for leukemia may have different short and long-term oral health effects of both disease and treatment compared to kids' w/ rhabdomyosarcoma)	We thank you for this valuable suggestion. We acknowledge that oral complications and their impact on quality of life may vary according to several factors, including the type of cancer, the child's age, baseline oral health status at diagnosis, and the type, dose, and modality of treatment, as well as the field of irradiation in the case of radiotherapy. While we recognize that different cancer diagnoses (e.g., leukemia versus	Line 72-75

	rhabdomyosarcoma) may be associated with distinct treatment pathways and may result in different oral health outcomes, the primary focus of this review is on treatment-related oral complications rather than diagnosis-specific effects. We have revised the introduction to explicitly clarify this scope and contextualize the variability across cancer therapies.	
Materials & Methods: Line 130 states "In cases of low agreement...reviewers adjusted eligibility criteria until substantial agreement was achieved." This was a bit confusing to me - would be helpful to provide additional context (i.e. whether this is a generally agreed-upon method, how any changes in eligibility criteria were applied across all considered reviews etc.)	Thank you for this comment. We agree that this section would benefit from additional clarification. The calibration exercise and iterative refinement of eligibility criteria are standard practices in systematic and scoping reviews to ensure consistent application of inclusion and exclusion criteria across reviewers. Any modifications made during this calibration phase were applied uniformly to all subsequent stages of screening. More description has been added to the manuscript as follows:<br>In cases of low agreement (<0.40), the reviewers discussed discrepancies and refined the eligibility criteria to improve clarity and consistency. This process was repeated until substantial agreement was achieved (>0.40). The final agreed-upon criteria were then applied uniformly to the screening of all remaining studies.	Line 152-155
Results: Consider including info about country of origin for each of the studies	Given the specific aims of our literature review, we did not plan to conduct analyses based on country of origin. Therefore, this variable was not included in this article.	
Results: Line 169: Consider summarizing the types of cancers included in this review (i.e. hematologic malignancies, sarcomas etc.) - this would be helpful for the reader to understand prognosis, treatments rendered (and likely oral health effects) etc. that may influence quality of life	Thank you for this valuable suggestion. The types of cancers included in the review have been summarized in the result section.	Line 198-203
Discussion: May want to use caution when directly comparing results of this review to the 2012 review, as it is not totally clear (at least to me) whether the methods were exactly the same	Thank you for this important comment. Our purpose was not to directly compare two reviews as equivalent studies, but to situate our new findings within the evolving literature and to highlight broad shifts in research focus and practice over time. To avoid any potential misinterpretation, we have revised the title of Table 3 and revised line 279 the Discussion.	Line 255, 279,

Discussion: Line 269: May want to elaborate more on long-term challenges among adult survivors of childhood cancer - could briefly discuss what has been found from studies focused on non-oral health effects	We thank the reviewer for this important suggestion. We have added additional details.	Line 325-330
Discussion: Line 275: May want to mention current professional guidelines (i.e. NCCN guidelines for follow-up care)	Thank you for this suggestion. While we acknowledge the relevance of professional guidelines in clinical management and follow-up of pediatric cancer survivors, the focus of this review is on summarizing the existing literature on oral health outcomes and quality of life rather than on guideline-based care recommendations. For this reason, we did not incorporate a discussion of current professional guidelines.	
Table 1: Consider placing this in the supplement and using this space for a summary table of the included studies	PRISMA checklist for reviews requires search strategy in the methodology section but we would be fine to move Table 1 to a supplement if the editor prefers this suggestion.<br>. We have revised Table 3 to serve both as a comparative and a summary table by reporting values as frequencies and percentages, informed by the data presented in Supplementary Table 1, rather than creating an additional table.	
Figure 1: Include an "n" for each of the excluded reasons	Included in Figure 1	
Table 3: May want to report as percentage or similar for comparison and include as a supplement, Was the OHIP-14 specific to children (citation 12)	Revised and percentage included in Table 3	
Supplemental Table 1:<br>- Consistent numbering (between S1 and text) or alphabetizing would be helpful for readers<br>- Explain n/a (assuming these were multiple cancer types?)<br>- Broadly, could report this info as a table in the main text	Thank you for these comments. A summary of the findings has been added to the main text. To improve clarity, n/a has been changed to NR (not recorded) to better fit the content. In addition, the numbering and labeling for Supplementary Table S1 has been organized alphabetically.	
**Reviewer 2**		
Keywords:<br>It is recommended that the keywords be revised to ensure alignment with official MeSH terms, thereby enhancing the indexing and discoverability of your empirical work. For instance, “Cancer treatment” does not appear to be a MeSH term. Additionally, given that the age range of children in the included studies is	The following keywords have been added: Neoplasm, Cancer survivors, Child, Adolescent, Therapeutics	

0-17 years, the keyword "Children" may not be the most appropriate keyword. "Children" is not a MeSH term as well. Please identify and substitute the closest corresponding MeSH equivalents to maintain consistency with indexing standards.		
Abstract: Line 25: In the Objective section, please consider adding the year of the previous review (e.g. "[2012]") to clearly indicate that the current review serves as an update to the earlier work.	We have added the year	Line 24
Abstract: Line 29: In the Methods section of the abstract, please consider specifying the electronic databases searched and noting that a predefined search strategy was used. If space permits, including the total number of records identified would strengthen the abstract. It would also be helpful to briefly mention the screening software used, that study selection was conducted by two independent reviewers with a third reviewer available to resolve disagreements, and that PRISMA-ScR guidelines were followed.	Thank you for your comments. These elements have been added.	Line 28-32
Abstract: Line 35: Please include the number of studies in parentheses (e.g., "…a substantial number of studies (XX) reported…").	The number has been included	Line 39
Abstract: Lines 35–36: Consider revising "children self-reported" to "children-reported" for improved clarity and coherence.	This has been revised	Line 40
Abstract: Line 38: Consider revising "use of measures of oral health-related quality of life (OHRQoL)…" to "reporting of oral health-related quality of life (OHRQoL) measures has increased" to improve clarity and flow.	This has been revised	Line 42
Abstract: Line 39: The current wording could be refined to more precisely reflect how frequently these measures were reported in the included literature. Rephrase "…however they were used in a minority of studies" to "however, they were observed in relatively few studies" or "reported in a limited number of studies."	This has been revised	Line 43

Abstract: Line 40: In the conclusion, consider revising "…growing recognition of the…" to a more action-oriented term (e.g., emphasis, adoption, implementation, or consideration), depending on what is best supported by the findings.	This has been revised	Line 44
Abstract: Line 42-44: The phrase "future direction" does not read clearly in this context and would benefit from revision. It is also unclear whether this statement refers to qualitative or mixed-methods assessments of children's experiences and perceptions of cancer therapy. If this is the intended meaning, please consider revising the sentence to: "Future studies should incorporate qualitative or mixed-methods approaches to capture children's or parents' perspectives on cancer therapy, thereby complementing quantitative findings and providing a more comprehensive understanding of the multifaceted impact of oral complications on children's quality of life."	This has been revised	Line 49-52
Introduction: Lines 83–84: Please add a citation to support the statement, "While having the parents' and health care providers' perspective is clearly important, it has become evident that children's perspectives are not always consistent with adults." In addition, briefly elaborating on how this inconsistency has been demonstrated (e.g., through empirical findings showing discordance between child- and adult-reported outcomes) would strengthen the argument and provide clearer context for readers.	This has been added	Line 100-101
Introduction: Line 90: The term "trend" may not be appropriate in this context, as the United Nations Convention on the Rights of the Child represents a normative framework or guiding principle rather than an observed trend. Please consider revising the sentence to reflect this distinction.	This has been revised	Line 107
Introduction: Line 92: The phrase "…since Noronha and Macdonald's review up to 2011"	This has been revised	Line 109

may be unclear to readers. For clarity, please consider specifying the publication year of that review (e.g., "published in 2012").		
Introduction: Lines 93–98: Consider numbering the stated aims of the review. This would help readers more easily align the results and discussion sections with the specific objectives outlined in the Introduction.	This has been revised	Line 133-138
Methods: Please include a brief description of the PRISMA-ScR framework, including eligibility and exclusion criteria, and the time frame covered by the literature search. While some of this information is mentioned in the abstract, it should be clearly documented within the Methods section itself to ensure methodological completeness.	This has been included	Line 133-138, 178-182
Results:<br>Lines 176–177: The term "well being" should be standardized throughout the manuscript. If a hyphen is used, please ensure consistent usage (e.g., "well-being" or "wellbeing") across the text.	This term has been standardized with a hyphen	
Discussion: Line 242: Consider replacing "children's voices" with "children's perceptions" to better align with the conceptual framing of the manuscript and maintain academic precision.	We have changed the phrase to "children's perspective"	Line 281
Discussion: Line 245: The term "concern" is vague in this context. Replacing it with "symptoms" would more accurately reflect the subjective experiences being discussed (e.g., pain, emotional distress, social functioning).	This has been revised	Line 284
Discussion: Line 254: Please add an appropriate citation to support the description of the assessment tools referenced in this statement.	This has been revised	Line 306
Discussion: Line 277: The statement regarding routine oral health assessments would benefit from clarification. Are such assessments not already part of standard pediatric oncology care? Please support this assertion with prior studies and cite evidence-based solutions where available.	Thank you for your feedback. We clarified the statement regarding routine oral health assessments and provided supporting evidence.	Line 334-337

Discussion: Line 278: Please consider revising the sentence "Additionally, psychosocial support should be integrated into care plans…" for clarity and conciseness. In addition, this recommendation should be supported with relevant citations demonstrating the psychosocial impact of visible dental anomalies or disfigurement in pediatric populations.	This has been revised	Line 340-342
Discussion: Lines 283–285: The recommendation for in-depth interviews and participatory research methods may not be feasible for younger children, particularly those aged 0–6 years and may also raise practical and ethical concerns (e.g., parental consent, cognitive ability to articulate experiences). Please consider revising this recommendation to reflect more age-appropriate qualitative or mixed-methods approaches.	We have revised this sentence.	Line 348-349
Conclusion: Line 287: Since the original review was published in 2012, please consider revising "In the last 14 years since the original review" to "In the 13 years since the original review" or "since the last review" for accuracy.	This has been revised	Line 365
Conclusion: Line 290: Please revise "children's self-reported symptoms" to "children-reported symptoms" for clarity and consistency.	This has been revised	Line 368
Conclusion: Lines 297–298: Consider strengthening this statement by explicitly incorporating the role of longitudinal research approaches (as discussed earlier around line 270). This addition would improve coherence between the Discussion and Conclusion sections.	The role of longitudinal research approaches has been added	Line 375-377
Additional suggestions: Consider reflecting on any unexpected findings, such as the relative distribution of study designs (e.g., fewer quantitative studies compared with qualitative studies), and discuss possible explanations in the context of existing literature.<br>Please add a brief statement outlining the strengths and limitations of this scoping review to enhance transparency.	Thank you for your comments. We added more discussion and have a limitation paragraph.	Line 290-300, 360-363

Consider expanding the policy implications by recommending country-level or system-level strategies (e.g., within the Canadian healthcare context) to support standardized data collection, integration of oral health metrics into pediatric oncology care, or inclusion of OHRQoL measures in routine practice. This would strengthen the discussion (Line 273-285)	Thank you for your practical comment. Relevant recommendations are added	Line 337-347, 351-359
