# Supplementary material for: The impact of cancer therapy-related oral complications on the quality of life and well-being of childhood cancer survivors: A scoping review
Source: PLoS One. 2026 Jun 23;21(6):e0352194. doi: 10.1371/journal.pone.0352194 (PMC13289919; doi:10.1371/journal.pone.0352194)
Supplement: Attachment — Submitted filename: Response_for_Reviewers_auresp_2.pdf [file pone.0352194.s007.pdf]

Response Letter

April 14, 2026

Giang Truong Vu,

Academic Editor

PLOS ONE

Subject: Submission of revised paper (PONE-D-25-18997)

Dear Academic Editor

Thank you for your email enclosing the reviewer's comments. We have carefully considered all comments and revised the manuscript accordingly. Our detailed responses are provided below in a point-by-point format.

Sincerely,

Nona Attaran

Corresponding author

**Comment**	**Response**	**Line# in tracked file**
Introduction: Thank you for the helpful updates. For additional clarity, I'd suggest including the years in which the cited studies were published, demonstrating changes since 2012. For example, something like "A 2007 study published by Hinds et al highlighted the value of child-focused PROs at end of life..." (Line 107).	Thank you for this comment. We agree that this section would benefit from additional clarification. Therefore, we revised the section to better demonstrate changes.	Line 102-111
Results: May want to comment on implications for generalizability in limitations	We thank you for this valuable suggestion a part focusing of generalizability has been added to the limitations	Line 384-387
Table 3: Thank you for the updates. I'd suggest including the date ranges for each study for further clarification. For example, original review (inclusive of years xx-2011 and current review (years 2012-2025).	Revised and date range has been included in the table title	Line 277-278
Supplemental Table 1: Thank you for the additions to this table. You may consider including the citation number from the main text in parentheses – For example, Okello DA article is citation 48 in the main text and 59 in Supplemental Table 1 with 59 in parentheses. In my opinion, would be clearer to have 48 in parentheses with a column heading titled "First Author (main text citation)" or similar	Thank you for the comment. A separate column with main text citation number has been added	S1Table
Line 45-46: ..."of the importance" extra words?	Thank you for the comment. Extra words are deleted.	Line 45-46
Line 215-220, 222-224, elsewhere as applicable: Consider adding percentages or number of articles for additional clarity – whichever is chosen, should be consistent throughout paper	Thank you for your comment. The number of articles are including.	Line 217,220,222, 223,229-234
Line 252: Do you mean statistical significance and association? If yes, consider reporting how association was measured – if not, consider a different word	Thank you for your comment. Wording has been changed for more clarification.	Line 257-258
Line 297: Did this shift yield different results?	Thank you for your comment. Wording has changed for more clarity	Line 317-318
Line 346: "Clear implications for clinical practice" may be too strong given that this was not explicitly analyzed in the review	Thank you for your comment. Wording has changed for more clarity	Line 354
Minor typographical and punctuation errors should be corrected	Thank you for your comment. The manuscript is reviewed for corrections.	
