# Supplementary material for: The impact of cancer therapy-related oral complications on the quality of life and well-being of childhood cancer survivors: A scoping review
Source: PLoS One. 2026 Jun 23;21(6):e0352194. doi: 10.1371/journal.pone.0352194 (PMC13289919; doi:10.1371/journal.pone.0352194)
Supplement: Attachment — Submitted filename: Review comments-12.24.25.docx [file pone.0352194.s003.docx]

Thank you for the opportunity to review the manuscript.

**Recommended changes/Modifications**-

**Keywords:**

It is recommended that the keywords be revised to ensure alignment with official MeSH terms, thereby enhancing the indexing and discoverability of your empirical work. For instance, “Cancer treatment” does not appear to be a MeSH term. Additionally, given that the age range of children in the included studies is 0-17 years, the keyword “Children” may not be the most appropriate keyword. “Children” is not a MeSH term as well. Please identify and substitute the closest corresponding MeSH equivalents to maintain consistency with indexing standards.

**Abstract**:

Line 25: In the Objective section, please consider adding the year of the previous review (e.g. “[2012]”) to clearly indicate that the current review serves as an update to the earlier work.

Line 29: In the Methods section of the abstract, please consider specifying the electronic databases searched and noting that a predefined search strategy was used. If space permits, including the total number of records identified would strengthen the abstract. It would also be helpful to briefly mention the screening software used, that study selection was conducted by two independent reviewers with a third reviewer available to resolve disagreements, and that PRISMA-ScR guidelines were followed.

Line 35: Please include the number of studies in parentheses (e.g., “…a substantial number of studies (XX) reported…”).

Lines 35–36: Consider revising “children self-reported” to “children-reported” for improved clarity and coherence.

Line 38: Consider revising “use of measures of oral health-related quality of life (OHRQoL)…” to “reporting of oral health-related quality of life (OHRQoL) measures has increased” to improve clarity and flow.

Line 39: The current wording could be refined to more precisely reflect how frequently these measures were reported in the included literature. Rephrase “…however they were used in a minority of studies” to “however, they were observed in relatively few studies” or “reported in a limited number of studies.”

Line 40: In the conclusion, consider revising “…growing recognition of the…” to a more action-oriented term (e.g., emphasis, adoption, implementation, or consideration), depending on what is best supported by the findings.

Line 42-44: The phrase “future direction” does not read clearly in this context and would benefit from revision. It is also unclear whether this statement refers to qualitative or mixed-methods assessments of children’s experiences and perceptions of cancer therapy. If this is the intended meaning, please consider revising the sentence to: “Future studies should incorporate qualitative or mixed-methods approaches to capture children’s or parents’ perspectives on cancer therapy, thereby complementing quantitative findings and providing a more comprehensive understanding of the multifaceted impact of oral complications on children’s quality of life.”

**Introduction**:

The section is well written overall; however, addressing the following points could further strengthen it:

Lines 83–84: Please add a citation to support the statement, “While having the parents’ and health care providers’ perspective is clearly important, it has become evident that children’s perspectives are not always consistent with adults.” In addition, briefly elaborating on how this inconsistency has been demonstrated (e.g., through empirical findings showing discordance between child- and adult-reported outcomes) would strengthen the argument and provide clearer context for readers.
Line 90: The term “trend” may not be appropriate in this context, as the United Nations Convention on the Rights of the Child represents a normative framework or guiding principle rather than an observed trend. Please consider revising the sentence to reflect this distinction.
Line 92: The phrase “…since Noronha and Macdonald’s review up to 2011” may be unclear to readers. For clarity, please consider specifying the publication year of that review (e.g., “published in 2012”).
Lines 93–98: Consider numbering the stated aims of the review. This would help readers more easily align the results and discussion sections with the specific objectives outlined in the Introduction.

**Methods**:

The Methods section is generally coherent and well organized, with clear subsections and logical flow. Addressing the following points would improve transparency and reproducibility:

Please include a brief description of the PRISMA-ScR framework, including eligibility and exclusion criteria, and the time frame covered by the literature search. While some of this information is mentioned in the abstract, it should be clearly documented within the Methods section itself to ensure methodological completeness.

**Results:**

The Results section is well drafted overall. The following minor revision would further improve clarity and consistency:

Lines 176–177: The term “well being” should be standardized throughout the manuscript. If a hyphen is used, please ensure consistent usage (e.g., “well-being” or “wellbeing”) across the text.

**Discussion**:

Overall, the Discussion section is well written and coherent. Addressing the minor points below and providing limited additional development—particularly through clearer contextualization of findings and selective engagement with existing literature—would further strengthen the section.

Line 242: Consider replacing “children’s voices” with “children’s perceptions” to better align with the conceptual framing of the manuscript and maintain academic precision.

Line 245: The term “concern” is vague in this context. Replacing it with “symptoms” would more accurately reflect the subjective experiences being discussed (e.g., pain, emotional distress, social functioning).

Line 254: Please add an appropriate citation to support the description of the assessment tools referenced in this statement.

Line 277: The statement regarding routine oral health assessments would benefit from clarification. Are such assessments not already part of standard pediatric oncology care? Please support this assertion with prior studies and cite evidence-based solutions where available.
Line 278: Please consider revising the sentence “Additionally, psychosocial support should be integrated into care plans…” for clarity and conciseness. In addition, this recommendation should be supported with relevant citations demonstrating the psychosocial impact of visible dental anomalies or disfigurement in pediatric populations.
Lines 283–285: The recommendation for in-depth interviews and participatory research methods may not be feasible for younger children, particularly those aged 0–6 years, and may also raise practical and ethical concerns (e.g., parental consent, cognitive ability to articulate experiences). Please consider revising this recommendation to reflect more age-appropriate qualitative or mixed-methods approaches.

**Conclusion:**
Line 287: Since the original review was published in 2012, please consider revising “In the last 14 years since the original review” to “In the 13 years since the original review” or “since the last review” for accuracy.
Line 290: Please revise “children’s self-reported symptoms” to “children-reported symptoms” for clarity and consistency.
Lines 297–298: Consider strengthening this statement by explicitly incorporating the role of longitudinal research approaches (as discussed earlier around line 270). This addition would improve coherence between the Discussion and Conclusion sections.

**Additional suggestions:**

Consider reflecting on any unexpected findings, such as the relative distribution of study designs (e.g., fewer quantitative studies compared with qualitative studies), and discuss possible explanations in the context of existing literature.

Please add a brief statement outlining the strengths and limitations of this scoping review to enhance transparency.

Consider expanding the policy implications by recommending country-level or system-level strategies (e.g., within the Canadian healthcare context) to support standardized data collection, integration of oral health metrics into pediatric oncology care, or inclusion of OHRQoL measures in routine practice. This would strengthen the discussion (Line 273-285)
