# Supplementary material for: The impact of cancer therapy-related oral complications on the quality of life and well-being of childhood cancer survivors: A scoping review
Source: PLoS One. 2026 Jun 23;21(6):e0352194. doi: 10.1371/journal.pone.0352194 (PMC13289919; doi:10.1371/journal.pone.0352194)
Supplement: Attachment — Submitted filename: PONE-D-25-18997_R1 Comments.docx [file pone.0352194.s005.docx]

**(1) Introduction: Would be helpful to provide very specific ways in which this update differs from the 2012 review**

Thank you for the helpful updates. For additional clarity, I’d suggest including the years in which the cited studies were published, demonstrating changes since 2012. For example, something like “A 2007 study published by Hinds et al highlighted the value of child-focused PROs at end of life...” (Line 107).

**(2) Results: Consider including info about country of origin for each of the studies**

May want to comment on implications for generalizability in limitations

**(3)** **Table 3.** Thank you for the updates. I’d suggest including the date ranges for each study for further clarification. For example, original review (inclusive of years xx-2011 and current review (years 2012-2025).

**(4) Supplemental Table 1.** Thank you for the additions to this table. You may consider including the citation number from the main text in parentheses – For example, Okello DA article is citation 48 in the main text and 59 in Supplemental Table 1 with 59 in parentheses. In my opinion, would be clearer to have 48 in parentheses with a column heading titled “First Author (main text citation)” or similar

**(5) Additional Comments:**

- Line 45-46: ...“of the importance” extra words?
- Line 215-220, 222-224, elsewhere as applicable: Consider adding percentages or number of articles for additional clarity – whichever is chosen, should be consistent throughout paper
- Line 252: Do you mean statistical significance and association? If yes, consider reporting how association was measured – if not, consider a different word
- Line 297: Did this shift yield different results?
- Line 346: “Clear implications for clinical practice” may be too strong given that this was not explicitly analyzed in the review
- Minor typographical and punctuation errors should be corrected
