# Supplementary material for: The impact of cancer therapy-related oral complications on the quality of life and well-being of childhood cancer survivors: A scoping review
Source: PLoS One. 2026 Jun 23;21(6):e0352194. doi: 10.1371/journal.pone.0352194 (PMC13289919; doi:10.1371/journal.pone.0352194)
Supplement: Peer review history

# Peer review: Decision Letter 0

**DOI:** 10.1371/journal.pone.0352194.r001

**Authors:** Giang Vu

9 Jan 2026

PONE-D-25-18997

The impact of cancer therapy-related oral complications on the quality of life and well-being of childhood cancer survivors: A scoping review

PLOS One

Dear Dr. Attaran,

Thank you for submitting your manuscript to PLOS ONE. After careful consideration, we feel that it has merit but does not fully meet PLOS ONE’s publication criteria as it currently stands. Therefore, we invite you to submit a revised version of the manuscript that addresses the points raised during the review process.

Please submit your revised manuscript by Feb 23 2026 11:59PM. If you will need more time than this to complete your revisions, please reply to this message or contact the journal office at `plosone@plos.org. When you’re ready to submit your revision, log on to https://www.editorialmanager.com/pone/ and select the ‘Submissions Needing Revision’ folder to locate your manuscript file.

Please include the following items when submitting your revised manuscript:

- A letter that responds to each point raised by the academic editor and reviewer(s). You should upload this letter as a separate file labeled ‘Response to Reviewers’.
- A marked-up copy of your manuscript that highlights changes made to the original version. You should upload this as a separate file labeled ‘Revised Manuscript with Track Changes’.
- An unmarked version of your revised paper without tracked changes. You should upload this as a separate file labeled ‘Manuscript’.

If you would like to make changes to your financial disclosure, please include your updated statement in your cover letter. Guidelines for resubmitting your figure files are available below the reviewer comments at the end of this letter.

If applicable, we recommend that you deposit your laboratory protocols in protocols.io to enhance the reproducibility of your results. Protocols.io assigns your protocol its own identifier (DOI) so that it can be cited independently in the future. For instructions see: https://journals.plos.org/plosone/s/submission-guidelines#loc-laboratory-protocols. Additionally, PLOS ONE offers an option for publishing peer-reviewed Lab Protocol articles, which describe protocols hosted on protocols.io. Read more information on sharing protocols at https://plos.org/protocols?utm_medium=editorial-email&utm_source=authorletters&utm_campaign=protocols.

We look forward to receiving your revised manuscript.

Kind regards,

Giang Truong Vu, D.D.S., M.S., Ph.D.

Academic Editor

PLOS One

**Journal Requirements:**

When submitting your revision, we need you to address these additional requirements.

1. Please ensure that your manuscript meets PLOS ONE’s style requirements, including those for file naming. The PLOS ONE style templates can be found at

https://journals.plos.org/plosone/s/file?id=wjVg/PLOSOne_formatting_sample_main_body.pdf and

https://journals.plos.org/plosone/s/file?id=ba62/PLOSOne_formatting_sample_title_authors_affiliations.pdf

2. Please note that PLOS One has specific guidelines on code sharing for submissions in which author-generated code underpins the findings in the manuscript. In these cases, we expect all author-generated code to be made available without restrictions upon publication of the work. Please review our guidelines at https://journals.plos.org/plosone/s/materials-and-software-sharing#loc-sharing-code and ensure that your code is shared in a way that follows best practice and facilitates reproducibility and reuse.

3. Please include captions for your Supporting Information files at the end of your manuscript, and update any in-text citations to match accordingly. Please see our Supporting Information guidelines for more information: http://journals.plos.org/plosone/s/supporting-information.

4. If the reviewer comments include a recommendation to cite specific previously published works, please review and evaluate these publications to determine whether they are relevant and should be cited. There is no requirement to cite these works unless the editor has indicated otherwise.

[Note: HTML markup is below. Please do not edit.]

Reviewers’ comments:

Reviewer’s Responses to Questions

**Comments to the Author**

1. Is the manuscript technically sound, and do the data support the conclusions?

The manuscript must describe a technically sound piece of scientific research with data that supports the conclusions. Experiments must have been conducted rigorously, with appropriate controls, replication, and sample sizes. The conclusions must be drawn appropriately based on the data presented.

Reviewer #1: Yes

Reviewer #2: Yes

**********

2. Has the statistical analysis been performed appropriately and rigorously?

Reviewer #1: N/A

Reviewer #2: Yes

**********

3. Have the authors made all data underlying the findings in their manuscript fully available?

The PLOS Data policy requires authors to make all data underlying the findings described in their manuscript fully available without restriction, with rare exception (please refer to the Data Availability Statement in the manuscript PDF file). The data should be provided as part of the manuscript or its supporting information, or deposited to a public repository. For example, in addition to summary statistics, the data points behind means, medians and variance measures should be available. If there are restrictions on publicly sharing data—e.g. participant privacy or use of data from a third party—those must be specified.

Reviewer #1: Yes

Reviewer #2: Yes

**********

4. Is the manuscript presented in an intelligible fashion and written in standard English?

PLOS ONE does not copyedit accepted manuscripts, so the language in submitted articles must be clear, correct, and unambiguous. Any typographical or grammatical errors should be corrected at revision, so please note any specific errors here.

Reviewer #1: Yes

Reviewer #2: Yes

**********

5. Review Comments to the Author

Please use the space provided to explain your answers to the questions above. You may also include additional comments for the author, including concerns about dual publication, research ethics, or publication ethics. (Please upload your review as an attachment if it exceeds 20,000 characters)

Reviewer #1: Thank you for engaging in this project. In summary, this is an update to a similar review published in 2012 by essentially the same research group. The authors seek to identify broad themes in the area of oral health-related quality life among children with cancer (including adult survivors). Suggestions are as follows:

(1) Abstract

- Include protocol registration

(2) Introduction

- Consider including the number of children living with cancer (preferably w/in the geographic area of the included articles), and the number of adult survivors of childhood cancers

- Line 49: Double-check whether statement "mortality rate continues to decrease" requires some nuance- I suspect this is the case for certain cancers in certain countries (i.e. leukemia in the high-resource countries) but not necessarily for other cancer types and in all resource settings

- Would be helpful to provide very specific ways in which this update differs from the 2012 review

- May want to elaborate more on the types of cancer therapies as how each may impact oral health and subsequently quality of life (for example, children treated for leukemia may have different short and long-term oral health effects of both disease and treatment compared to kids w/ rhabdomyosarcoma)

(2) Materials & Methods

- Line 130 states "In cases of low agreement...reviewers adjusted eligibility criteria until substantial agreement was achieved." This was a bit confusing to me - would be helpful to provide additional context (i.e. whether this is a generally agreed-upon method, how any changes in eligibility criteria were applied across all considered reviews etc.)

(3) Results

- Consider including info about country of origin for each of the studies

- Line 169: Consider summarizing the types of cancers included in this review (i.e. hematologic malignancies, sarcomas etc.) - this would be helpful for the reader to understand prognosis, treatments rendered (and likely oral health effects) etc. that may influence quality of life

(4) Discussion

- May want to use caution when directly comparing results of this review of to the 2012 review, as it is not totally clear (at least to me) whether the methods were exactly the same

- Line 269: May want to elaborate more on long-term challenges among adult survivors of childhood cancer - could briefly discuss what has been found from studies focused on non-oral health effects

- Line 275: May want to mention current professional guidelines (i.e. NCCN guidelines for follow-up care)

(5) Tables & Figures:

Table 1:

- Consider placing this in the supplement and using this space for a summary table of the included studies

Figure 1:

- Include an "n" for each of the excluded reasons

Figure 2:

- Correct spelling and capitalization errors

Table 3:

- May want to report as percentage or similar for comparison and include as a supplement

- Was the OHIP-14 specific to children (citation 12)?

Supplemental Table 1:

- Consistent numbering (between S1 and text) or alphabetizing would be helpful for readers

- Explain n/a (assuming these were multiple cancer types?)

- Broadly, could report this info as a table in the main text

Reviewer #2: Thank you for the opportunity to review the manuscript.

Recommended changes/Modifications-

Keywords:

It is recommended that the keywords be revised to ensure alignment with official MeSH terms, thereby enhancing the indexing and discoverability of your empirical work. For instance, “Cancer treatment” does not appear to be a MeSH term. Additionally, given that the age range of children in the included studies is 0-17 years, the keyword “Children” may not be the most appropriate keyword. “Children” is not a MeSH term as well. Please identify and substitute the closest corresponding MeSH equivalents to maintain consistency with indexing standards.

Abstract:

Line 25: In the Objective section, please consider adding the year of the previous review (e.g. “[2012]”) to clearly indicate that the current review serves as an update to the earlier work.

Line 29: In the Methods section of the abstract, please consider specifying the electronic databases searched and noting that a predefined search strategy was used. If space permits, including the total number of records identified would strengthen the abstract. It would also be helpful to briefly mention the screening software used, that study selection was conducted by two independent reviewers with a third reviewer available to resolve disagreements, and that PRISMA-ScR guidelines were followed.

Line 35: Please include the number of studies in parentheses (e.g., “…a substantial number of studies (XX) reported…”).

Lines 35–36: Consider revising “children self-reported” to “children-reported” for improved clarity and coherence.

Line 38: Consider revising “use of measures of oral health-related quality of life (OHRQoL)…” to “reporting of oral health-related quality of life (OHRQoL) measures has increased” to improve clarity and flow.

Line 39: The current wording could be refined to more precisely reflect how frequently these measures were reported in the included literature. Rephrase “…however they were used in a minority of studies” to “however, they were observed in relatively few studies” or “reported in a limited number of studies.”

Line 40: In the conclusion, consider revising “…growing recognition of the…” to a more action-oriented term (e.g., emphasis, adoption, implementation, or consideration), depending on what is best supported by the findings.

Line 42-44: The phrase “future direction” does not read clearly in this context and would benefit from revision. It is also unclear whether this statement refers to qualitative or mixed-methods assessments of children’s experiences and perceptions of cancer therapy. If this is the intended meaning, please consider revising the sentence to: “Future studies should incorporate qualitative or mixed-methods approaches to capture children’s or parents’ perspectives on cancer therapy, thereby complementing quantitative findings and providing a more comprehensive understanding of the multifaceted impact of oral complications on children’s quality of life.”

Introduction:

The section is well written overall; however, addressing the following points could further strengthen it:

Lines 83–84: Please add a citation to support the statement, “While having the parents’ and health care providers’ perspective is clearly important, it has become evident that children’s perspectives are not always consistent with adults.” In addition, briefly elaborating on how this inconsistency has been demonstrated (e.g., through empirical findings showing discordance between child- and adult-reported outcomes) would strengthen the argument and provide clearer context for readers.

Line 90: The term “trend” may not be appropriate in this context, as the United Nations Convention on the Rights of the Child represents a normative framework or guiding principle rather than an observed trend. Please consider revising the sentence to reflect this distinction.

Line 92: The phrase “…since Noronha and Macdonald’s review up to 2011” may be unclear to readers. For clarity, please consider specifying the publication year of that review (e.g., “published in 2012”).

Lines 93–98: Consider numbering the stated aims of the review. This would help readers more easily align the results and discussion sections with the specific objectives outlined in the Introduction.

Methods:

The Methods section is generally coherent and well organized, with clear subsections and logical flow. Addressing the following points would improve transparency and reproducibility:

Please include a brief description of the PRISMA-ScR framework, including eligibility and exclusion criteria, and the time frame covered by the literature search. While some of this information is mentioned in the abstract, it should be clearly documented within the Methods section itself to ensure methodological completeness.

Results:

The Results section is well drafted overall. The following minor revision would further improve clarity and consistency:

Lines 176–177: The term “well being” should be standardized throughout the manuscript. If a hyphen is used, please ensure consistent usage (e.g., “well-being” or “wellbeing”) across the text.

Discussion:

Overall, the Discussion section is well written and coherent. Addressing the minor points below and providing limited additional development—particularly through clearer contextualization of findings and selective engagement with existing literature—would further strengthen the section.

Line 242: Consider replacing “children’s voices” with “children’s perceptions” to better align with the conceptual framing of the manuscript and maintain academic precision.

Line 245: The term “concern” is vague in this context. Replacing it with “symptoms” would more accurately reflect the subjective experiences being discussed (e.g., pain, emotional distress, social functioning).

Line 254: Please add an appropriate citation to support the description of the assessment tools referenced in this statement.

Line 277: The statement regarding routine oral health assessments would benefit from clarification. Are such assessments not already part of standard pediatric oncology care? Please support this assertion with prior studies and cite evidence-based solutions where available.

Line 278: Please consider revising the sentence “Additionally, psychosocial support should be integrated into care plans…” for clarity and conciseness. In addition, this recommendation should be supported with relevant citations demonstrating the psychosocial impact of visible dental anomalies or disfigurement in pediatric populations.

Lines 283–285: The recommendation for in-depth interviews and participatory research methods may not be feasible for younger children, particularly those aged 0–6 years, and may also raise practical and ethical concerns (e.g., parental consent, cognitive ability to articulate experiences). Please consider revising this recommendation to reflect more age-appropriate qualitative or mixed-methods approaches.

Conclusion:

Line 287: Since the original review was published in 2012, please consider revising “In the last 14 years since the original review” to “In the 13 years since the original review” or “since the last review” for accuracy.

Line 290: Please revise “children’s self-reported symptoms” to “children-reported symptoms” for clarity and consistency.

Lines 297–298: Consider strengthening this statement by explicitly incorporating the role of longitudinal research approaches (as discussed earlier around line 270). This addition would improve coherence between the Discussion and Conclusion sections.

Additional suggestions:

Consider reflecting on any unexpected findings, such as the relative distribution of study designs (e.g., fewer quantitative studies compared with qualitative studies), and discuss possible explanations in the context of existing literature.

Please add a brief statement outlining the strengths and limitations of this scoping review to enhance transparency.

Consider expanding the policy implications by recommending country-level or system-level strategies (e.g., within the Canadian healthcare context) to support standardized data collection, integration of oral health metrics into pediatric oncology care, or inclusion of OHRQoL measures in routine practice. This would strengthen the discussion (Line 273-285)

**********

6. PLOS authors have the option to publish the peer review history of their article (what does this mean?). If published, this will include your full peer review and any attached files.

If you choose “no”, your identity will remain anonymous but your review may still be made public.

**Do you want your identity to be public for this peer review?**  For information about this choice, including consent withdrawal, please see our Privacy Policy.

Reviewer #1: **Yes:** Leah I. Leinbach DMD MPH

Reviewer #2: No

**********

[NOTE: If reviewer comments were submitted as an attachment file, they will be attached to this email and accessible via the submission site. Please log into your account, locate the manuscript record, and check for the action link "View Attachments". If this link does not appear, there are no attachment files.]

To ensure your figures meet our technical requirements, please review our figure guidelines: https://journals.plos.org/plosone/s/figures

You may also use PLOS’s free figure tool, NAAS, to help you prepare publication quality figures: https://journals.plos.org/plosone/s/figures#loc-tools-for-figure-preparation.

NAAS will assess whether your figures meet our technical requirements by comparing each figure against our figure specifications.

Attachment Submitted filename: Review comments-12.24.25.docx

# Author comment: Author response to Decision Letter 1

**DOI:** 10.1371/journal.pone.0352194.r002

18 Feb 2026

Response Letter

Feb 2, 2026

Giang Truong Vu,

Academic Editor

PLOS ONE

Subject: Submission of revised paper (PONE-D-25-18997)

Dear Academic Editor

Thank you for your email enclosing the reviewers’ comments. We have carefully considered all comments and revised the manuscript accordingly. Our detailed responses are provided below in a point-by-point format.

Sincerely,

Nona Attaran

Corresponding author

Comment Response Line# in tracked file

Reviewer 1

Abstract: Include protocol registration The protocol has been included in the abstract. Line 36-37

Introduction: Consider including the number of children living with cancer (preferably w/in the geographic area of the included articles), and the number of adult survivors of childhood cancers We have included the estimated number of new children diagnosed with cancer in a five-year rolling period according to the latest update of the Public Health Agency of Canada on cancer in children in Canada. Note: we could not locate a reference for adult survivors. Line 55-57

Introduction: Line 49: Double-check whether statement "mortality rate continues to decrease" requires some nuance- I suspect this is the case for certain cancers in certain countries (i.e. leukemia in the high-resource countries) but not necessarily for other cancer types and in all resource settings

Thank you for highlighting this important nuance. We agree that childhood cancer mortality remains disproportionately high in low-income countries. Our original statement was intended to reflect the overall global trend, which indicates a decrease in childhood cancer mortality over the past decade. In response to this comment, we have revised the paragraph to explicitly acknowledge this disparity and clarify the distinction between global trends and regional rates.

Line 57-60

Introduction: Would be helpful to provide very specific ways in which this update differs from the 2012 review

We are unclear how to better outline this section. As we mentioned already, this review adds three new dimensions to the 2012 review, as follows:

1. We have added whether (and how) there has been increased attention to OHRQoL.

2. We expanded the scope of the study to include adult survivors of childhood cancer in order to capture the long-term impact of cancer treatment complications on quality of life and well-being.

3. We focused on if and how research approaches increasingly acknowledge and incorporate the unique insights, experiences, and perspectives of children surviving cancer on their quality of life and well-being.

Please advise if this is insufficient.

Line 85-116

Introduction: May want to elaborate more on the types of cancer therapies as how each may impact oral health and subsequently quality of life (for example, children treated for leukemia may have different short and long-term oral health effects of both disease and treatment compared to kids’ w/ rhabdomyosarcoma) We thank you for this valuable suggestion. We acknowledge that oral complications and their impact on quality of life may vary according to several factors, including the type of cancer, the child’s age, baseline oral health status at diagnosis, and the type, dose, and modality of treatment, as well as the field of irradiation in the case of radiotherapy. While we recognize that different cancer diagnoses (e.g., leukemia versus rhabdomyosarcoma) may be associated with distinct treatment pathways and may result in different oral health outcomes, the primary focus of this review is on treatment-related oral complications rather than diagnosis-specific effects. We have revised the introduction to explicitly clarify this scope and contextualize the variability across cancer therapies.

Line 72-75

Materials & Methods: Line 130 states "In cases of low agreement...reviewers adjusted eligibility criteria until substantial agreement was achieved." This was a bit confusing to me - would be helpful to provide additional context (i.e. whether this is a generally agreed-upon method, how any changes in eligibility criteria were applied across all considered reviews etc.)

Thank you for this comment. We agree that this section would benefit from additional clarification. The calibration exercise and iterative refinement of eligibility criteria are standard practices in systematic and scoping reviews to ensure consistent application of inclusion and exclusion criteria across reviewers. Any modifications made during this calibration phase were applied uniformly to all subsequent stages of screening. More description has been added to the manuscript as follows:

In cases of low agreement (<0.40), the reviewers discussed discrepancies and refined the eligibility criteria to improve clarity and consistency. This process was repeated until substantial agreement was achieved (>0.40). The final agreed-upon criteria were then applied uniformly to the screening of all remaining studies. Line 152-155

Results: Consider including info about country of origin for each of the studies Given the specific aims of our literature review, we did not plan to conduct analyses based on country of origin. Therefore, this variable was not included in this article.

Results: Line 169: Consider summarizing the types of cancers included in this review (i.e. hematologic malignancies, sarcomas etc.) - this would be helpful for the reader to understand prognosis, treatments rendered (and likely oral health effects) etc. that may influence quality of life Thank you for this valuable suggestion. The types of cancers included in the review have been summarized in the result section. Line 198-203

Discussion: May want to use caution when directly comparing results of this review to the 2012 review, as it is not totally clear (at least to me) whether the methods were exactly the same

Thank you for this important comment. Our purpose was not to directly compare two reviews as equivalent studies, but to situate our new findings within the evolving literature and to highlight broad shifts in research focus and practice over time. To avoid any potential misinterpretation, we have revised the title of Table 3 and revised line 279 the Discussion.

Line 255, 279,

Discussion: Line 269: May want to elaborate more on long-term challenges among adult survivors of childhood cancer - could briefly discuss what has been found from studies focused on non-oral health effects We thank the reviewer for this important suggestion. We have added additional details.

Line 325-330

Discussion: Line 275: May want to mention current professional guidelines (i.e. NCCN guidelines for follow-up care) Thank you for this suggestion. While we acknowledge the relevance of professional guidelines in clinical management and follow-up of pediatric cancer survivors, the focus of this review is on summarizing the existing literature on oral health outcomes and quality of life rather than on guideline-based care recommendations. For this reason, we did not incorporate a discussion of current professional guidelines.

Table 1: Consider placing this in the supplement and using this space for a summary table of the included studies

PRISMA checklist for reviews requires search strategy in the methodology section but we would be fine to move Table 1 to a supplement if the editor prefers this suggestion.

We have revised Table 3 to serve both as a comparative and a summary table by reporting values as frequencies and percentages, informed by the data presented in Supplementary Table 1, rather than creating an additional table.

Figure 1: Include an "n" for each of the excluded reasons Included in Figure 1

Table 3: May want to report as percentage or similar for comparison and include as a supplement, Was the OHIP-14 specific to children (citation 12)

Revised and percentage included in Table 3

Supplemental Table 1:

- Consistent numbering (between S1 and text) or alphabetizing would be helpful for readers

- Explain n/a (assuming these were multiple cancer types?)

- Broadly, could report this info as a table in the main text Thank you for these comments. A summary of the findings has been added to the main text. To improve clarity, n/a has been changed to NR (not recorded) to better fit the content. In addition, the numbering and labeling for Supplementary Table S1 has been organized alphabetically.

Reviewer 2

Keywords:

It is recommended that the keywords be revised to ensure alignment with official MeSH terms, thereby enhancing the indexing and discoverability of your empirical work. For instance, “Cancer treatment” does not appear to be a MeSH term. Additionally, given that the age range of children in the included studies is 0-17 years, the keyword “Children” may not be the most appropriate keyword. “Children” is not a MeSH term as well. Please identify and substitute the closest corresponding MeSH equivalents to maintain consistency with indexing standards.

The following keywords have been added: Neoplasm, Cancer survivors, Child, Adolescent, Therapeutics

Abstract: Line 25: In the Objective section, please consider adding the year of the previous review (e.g. “[2012]”) to clearly indicate that the current review serves as an update to the earlier work.

We have added the year Line 24

Abstract: Line 29: In the Methods section of the abstract, please consider specifying the electronic databases searched and noting that a predefined search strategy was used. If space permits, including the total number of records identified would strengthen the abstract. It would also be helpful to briefly mention the screening software used, that study selection was conducted by two independent reviewers with a third reviewer available to resolve disagreements, and that PRISMA-ScR guidelines were followed.

Thank you for your comments. These elements have been added. Line 28-32

Abstract: Line 35: Please include the number of studies in parentheses (e.g., “…a substantial number of studies (XX) reported…”).

The number has been included Line 39

Abstract: Lines 35–36: Consider revising “children self-reported” to “children-reported” for improved clarity and coherence.

This has been revised Line 40

Abstract: Line 38: Consider revising “use of measures of oral health-related quality of life (OHRQoL)…” to “reporting of oral health-related quality of life (OHRQoL) measures has increased” to improve clarity and flow.

This has been revised Line 42

Abstract: Line 39: The current wording could be refined to more precisely reflect how frequently these measures were reported in the included literature. Rephrase “…however they were used in a minority of studies” to “however, they were observed in relatively few studies” or “reported in a limited number of studies.”

This has been revised Line 43

Abstract: Line 40: In the conclusion, consider revising “…growing recognition of the…” to a more action-oriented term (e.g., emphasis, adoption, implementation, or consideration), depending on what is best supported by the findings.

This has been revised Line 44

Abstract: Line 42-44: The phrase “future direction” does not read clearly in this context and would benefit from revision. It is also unclear whether this statement refers to qualitative or mixed-methods assessments of children’s experiences and perceptions of cancer therapy. If this is the intended meaning, please consider revising the sentence to: “Future studies should incorporate qualitative or mixed-methods approaches to capture children’s or parents’ perspectives on cancer therapy, thereby complementing quantitative findings and providing a more comprehensive understanding of the multifaceted impact of oral complications on children’s quality of life.”

This has been revised Line 49-52

Introduction: Lines 83–84: Please add a citation to support the statement, “While having the parents’ and health care providers’ perspective is clearly important, it has become evident that children’s perspectives are not always consistent with adults.” In addition, briefly elaborating on how this inconsistency has been demonstrated (e.g., through empirical findings showing discordance between child- and adult-reported outcomes) would strengthen the argument and provide clearer context for readers.

This has been added Line 100-101

Introduction: Line 90: The term “trend” may not be appropriate in this context, as the United Nations Convention on the Rights of the Child represents a normative framework or guiding principle rather than an observed trend. Please consider revising the sentence to reflect this distinction.

This has been revised Line 107

Introduction: Line 92: The phrase “…since Noronha and Macdonald’s review up to 2011” may be unclear to readers. For clarity, please consider specifying the publication year of that review (e.g., “published in 2012”).

This has been revised Line 109

Introduction: Lines 93–98: Consider numbering the stated aims of the review. This would help readers more easily align the results and discussion sections with the specific objectives outlined in the Introduction. This has been revised Line 133-138

Methods: Please include a brief description of the PRISMA-ScR framework, including eligibility and exclusion criteria, and the time frame covered by the literature search. While some of this information is mentioned in the abstract, it should be clearly documented within the Methods section itself to ensure methodological completeness.

This has been included Line 133-138, 178-182

Results:

Lines 176–177: The term “well being” should be standardized throughout the manuscript. If a hyphen is used, please ensure consistent usage (e.g., “well-being” or “wellbeing”) across the text.

This term has been standardized with a hyphen

Discussion: Line 242: Consider replacing “children’s voices” with “children’s perceptions” to better align with the conceptual framing of the manuscript and maintain academic precision. We have changed the phrase to “children’s perspective” Line 281

Discussion: Line 245: The term “concern” is vague in this context. Replacing it with “symptoms” would more accurately reflect the subjective experiences being discussed (e.g., pain, emotional distress, social functioning).

This has been revised Line 284

Discussion: Line 254: Please add an appropriate citation to support the description of the assessment tools referenced in this statement.

This has been revised Line 306

Discussion: Line 277: The statement regarding routine oral health assessments would benefit from clarification. Are such assessments not already part of standard pediatric oncology care? Please support this assertion with prior studies and cite evidence-based solutions where available. Thank you for your feedback. We clarified the statement regarding routine oral health assessments and provided supporting evidence. Line 334-337

Discussion: Line 278: Please consider revising the sentence “Additionally, psychosocial support should be integrated into care plans…” for clarity and conciseness. In addition, this recommendation should be supported with relevant citations demonstrating the psychosocial impact of visible dental anomalies or disfigurement in pediatric populations.

This has been revised Line 340-342

Discussion: Lines 283–285: The recommendation for in-depth interviews and participatory research methods may not be feasible for younger children, particularly those aged 0–6 years and may also raise practical and ethical concerns (e.g., parental consent, cognitive ability to articulate experiences). Please consider revising this recommendation to reflect more age-appropriate qualitative or mixed-methods approaches.

We have revised this sentence. Line 348-349

Conclusion: Line 287: Since the original review was published in 2012, please consider revising “In the last 14 years since the original review” to “In the 13 years since the original review” or “since the last review” for accuracy. This has been revised Line 365

Conclusion: Line 290: Please revise “children’s self-reported symptoms” to “children-reported symptoms” for clarity and consistency.

This has been revised Line 368

Conclusion: Lines 297–298: Consider strengthening this statement by explicitly incorporating the role of longitudinal research approaches (as discusse

Attachment Submitted filename: Response for Reviewers.pdf

# Peer review: Decision Letter 1

**DOI:** 10.1371/journal.pone.0352194.r003

**Authors:** Giang Vu, Giang Vu

9 Apr 2026

PONE-D-25-18997R1

The impact of cancer therapy-related oral complications on the quality of life and well-being of childhood cancer survivors: A scoping review

PLOS One

Dear Dr. Attaran,

Thank you for submitting your manuscript to PLOS ONE. After careful consideration, we feel that it has merit but does not fully meet PLOS ONE’s publication criteria as it currently stands. Therefore, we invite you to submit a revised version of the manuscript that addresses the points raised during the review process.

Please submit your revised manuscript by May 24 2026 11:59PM. If you will need more time than this to complete your revisions, please reply to this message or contact the journal office at `plosone@plos.org. When you’re ready to submit your revision, log on to https://www.editorialmanager.com/pone/ and select the ‘Submissions Needing Revision’ folder to locate your manuscript file.

Please include the following items when submitting your revised manuscript:

- A letter that responds to each point raised by the academic editor and reviewer(s). You should upload this letter as a separate file labeled ‘Response to Reviewers’.
- A marked-up copy of your manuscript that highlights changes made to the original version. You should upload this as a separate file labeled ‘Revised Manuscript with Track Changes’.
- An unmarked version of your revised paper without tracked changes. You should upload this as a separate file labeled ‘Manuscript’.

If you would like to make changes to your financial disclosure, please include your updated statement in your cover letter. Guidelines for resubmitting your figure files are available below the reviewer comments at the end of this letter.

If applicable, we recommend that you deposit your laboratory protocols in protocols.io to enhance the reproducibility of your results. Protocols.io assigns your protocol its own identifier (DOI) so that it can be cited independently in the future. For instructions see: https://journals.plos.org/plosone/s/submission-guidelines#loc-laboratory-protocols. Additionally, PLOS ONE offers an option for publishing peer-reviewed Lab Protocol articles, which describe protocols hosted on protocols.io. Read more information on sharing protocols at https://plos.org/protocols?utm_medium=editorial-email&utm_source=authorletters&utm_campaign=protocols.

As the corresponding author, your ORCID iD is verified in the submission system and will appear in the published article. PLOS supports the use of ORCID, and we encourage all coauthors to register for an ORCID iD and use it as well. Please encourage your coauthors to verify their ORCID iD within the submission system before final acceptance, as unverified ORCID iDs will not appear in the published article. *Only*  the individual author can complete the verification step; PLOS staff *cannot*  verify ORCID iDs on behalf of authors.

We look forward to receiving your revised manuscript.

Kind regards,

Giang Vu, D.D.S., M.S., Ph.D.

Academic Editor

PLOS One

Reviewers’ comments:

Reviewer’s Responses to Questions

**Comments to the Author**

1. If the authors have adequately addressed your comments raised in a previous round of review and you feel that this manuscript is now acceptable for publication, you may indicate that here to bypass the “Comments to the Author” section, enter your conflict of interest statement in the “Confidential to Editor” section, and submit your "Accept" recommendation.

Reviewer #2: All comments have been addressed

Reviewer #3: (No Response)

**********

2. Is the manuscript technically sound, and do the data support the conclusions?

The manuscript must describe a technically sound piece of scientific research with data that supports the conclusions. Experiments must have been conducted rigorously, with appropriate controls, replication, and sample sizes. The conclusions must be drawn appropriately based on the data presented.

Reviewer #2: Yes

Reviewer #3: Yes

**********

3. Has the statistical analysis been performed appropriately and rigorously?

Reviewer #2: Yes

Reviewer #3: N/A

**********

4. Have the authors made all data underlying the findings in their manuscript fully available?

The PLOS Data policy requires authors to make all data underlying the findings described in their manuscript fully available without restriction, with rare exception (please refer to the Data Availability Statement in the manuscript PDF file). The data should be provided as part of the manuscript or its supporting information, or deposited to a public repository. For example, in addition to summary statistics, the data points behind means, medians and variance measures should be available. If there are restrictions on publicly sharing data—e.g. participant privacy or use of data from a third party—those must be specified.

Reviewer #2: Yes

Reviewer #3: Yes

**********

5. Is the manuscript presented in an intelligible fashion and written in standard English?

PLOS ONE does not copyedit accepted manuscripts, so the language in submitted articles must be clear, correct, and unambiguous. Any typographical or grammatical errors should be corrected at revision, so please note any specific errors here.

Reviewer #2: Yes

Reviewer #3: No

**********

6. Review Comments to the Author

Please use the space provided to explain your answers to the questions above. You may also include additional comments for the author, including concerns about dual publication, research ethics, or publication ethics. (Please upload your review as an attachment if it exceeds 20,000 characters)

Reviewer #2: The authors have thoroughly addressed all reviewer comments, and the revisions have significantly strengthened the manuscript. The paper is well-written and clear. I recommend it for publication.

Reviewer #3: Overall, the manuscript has been strengthened since last revision. Below are some minor recommendations:

(1) Introduction: Would be helpful to provide very specific ways in which this update differs from the 2012 review

Thank you for the helpful updates. For additional clarity, I’d suggest including the years in which the cited studies were published, demonstrating changes since 2012. For example, something like “A 2007 study published by Hinds et al highlighted the value of child-focused PROs at end of life...” (Line 107).

(2) Results: Consider including info about country of origin for each of the studies

May want to comment on implications for generalizability in limitations

(3) Table 3. Thank you for the updates. I’d suggest including the date ranges for each study for further clarification. For example, original review (inclusive of years xx-2011 and current review (years 2012-2025).

(4) Supplemental Table 1. Thank you for the additions to this table. You may consider including the citation number from the main text in parentheses – For example, Okello DA article is citation 48 in the main text and 59 in Supplemental Table 1 with 59 in parentheses. In my opinion, would be clearer to have 48 in parentheses with a column heading titled “First Author (main text citation)” or similar

(5) Additional Comments:

Line 45-46: ...“of the importance” extra words?

Line 215-220, 222-224, elsewhere as applicable: Consider adding percentages or number of articles for additional clarity – whichever is chosen, should be consistent throughout paper

Line 252: Do you mean statistical significance and association? If yes, consider reporting how association was measured – if not, consider a different word

Line 297: Did this shift yield different results?

Line 346: “Clear implications for clinical practice” may be too strong given that this was not explicitly analyzed in the review

Minor typographical and punctuation errors should be corrected

**********

7. PLOS authors have the option to publish the peer review history of their article (what does this mean?). If published, this will include your full peer review and any attached files.

If you choose “no”, your identity will remain anonymous but your review may still be made public.

**Do you want your identity to be public for this peer review?**  For information about this choice, including consent withdrawal, please see our Privacy Policy.

Reviewer #2: **Yes:** Bedant Chakraborty

Reviewer #3: **Yes:** Leah Leinbach

**********

To ensure your figures meet our technical requirements, please review our figure guidelines: https://journals.plos.org/plosone/s/figures

You may also use PLOS’s free figure tool, NAAS, to help you prepare publication quality figures: https://journals.plos.org/plosone/s/figures#loc-tools-for-figure-preparation.

NAAS will assess whether your figures meet our technical requirements by comparing each figure against our figure specifications.

Attachment Submitted filename: PONE-D-25-18997_R1 Comments.docx

# Author comment: Author response to Decision Letter 2

**DOI:** 10.1371/journal.pone.0352194.r004

18 May 2026

Response Letter

April 14, 2026

Giang Truong Vu,

Academic Editor

PLOS ONE

Subject: Submission of revised paper (PONE-D-25-18997)

Dear Academic Editor

Thank you for your email enclosing the reviewer’s comments. We have carefully considered all comments and revised the manuscript accordingly. Our detailed responses are provided below in a point-by-point format.

Sincerely,

Nona Attaran

Corresponding author

Comment Response Line# in tracked file

Introduction: Thank you for the helpful updates. For additional clarity, I’d suggest including the years in which the cited studies were published, demonstrating changes since 2012. For example, something like “A 2007 study published by Hinds et al highlighted the value of child-focused PROs at end of life...” (Line 107). Thank you for this comment. We agree that this section would benefit from additional clarification. Therefore, we revised the section to better demonstrate changes. Line 102-111

Results: May want to comment on implications for generalizability in limitations We thank you for this valuable suggestion a part focusing of generalizability has been added to the limitations Line 384-387

Table 3: Thank you for the updates. I’d suggest including the date ranges for each study for further clarification. For example, original review (inclusive of years xx-2011 and current review (years 2012-2025). Revised and date range has been included in the table title Line 277-278

Supplemental Table 1: Thank you for the additions to this table. You may consider including the citation number from the main text in parentheses – For example, Okello DA article is citation 48 in the main text and 59 in Supplemental Table 1 with 59 in parentheses. In my opinion, would be clearer to have 48 in parentheses with a column heading titled “First Author (main text citation)” or similar Thank you for the comment. A separate column with main text citation number has been added

S1Table

Line 45-46: ...“of the importance” extra words? Thank you for the comment. Extra words are deleted. Line 45-46

Line 215-220, 222-224, elsewhere as applicable: Consider adding percentages or number of articles for additional clarity – whichever is chosen, should be consistent throughout paper Thank you for your comment. The number of articles are including. Line 217,220,222,

223,229-234

Line 252: Do you mean statistical significance and association? If yes, consider reporting how association was measured – if not, consider a different word Thank you for your comment. Wording has been changed for more clarification. Line 257-258

Line 297: Did this shift yield different results? Thank you for your comment. Wording has changed for more clarity Line 317-318

Line 346: “Clear implications for clinical practice” may be too strong given that this was not explicitly analyzed in the review Thank you for your comment. Wording has changed for more clarity Line 354

Minor typographical and punctuation errors should be corrected Thank you for your comment. The manuscript is reviewed for corrections.

Attachment Submitted filename: Response_for_Reviewers_auresp_2.pdf

# Peer review: Decision Letter 2

**DOI:** 10.1371/journal.pone.0352194.r005

**Authors:** Giang Vu, Giang Vu, Giang Vu

7 Jun 2026

The impact of cancer therapy-related oral complications on the quality of life and well-being of childhood cancer survivors: A scoping review

PONE-D-25-18997R2

Dear Dr. Attaran,

We’re pleased to inform you that your manuscript has been judged scientifically suitable for publication and will be formally accepted for publication once it meets all outstanding technical requirements.

Within one week, you’ll receive an e-mail detailing the required amendments. When these have been addressed, you’ll receive a formal acceptance letter and your manuscript will be scheduled for publication.

An invoice will be generated when your article is formally accepted. Please note, if your institution has a publishing partnership with PLOS and your article meets the relevant criteria, all or part of your publication costs will be covered. Please make sure your user information is up-to-date by logging into Editorial Manager at Editorial Manager® and clicking the ‘Update My Information’ link at the top of the page. For questions related to billing, please contact billing support.

If your institution or institutions have a press office, please notify them about your upcoming paper to help maximize its impact. If they’ll be preparing press materials, please inform our press team as soon as possible -- no later than 48 hours after receiving the formal acceptance. Your manuscript will remain under strict press embargo until 2 pm Eastern Time on the date of publication. For more information, please contact onepress@plos.org.

Kind regards,

Giang Vu, D.D.S., M.S., Ph.D.

Academic Editor

PLOS One

Reviewers’ comments:

Reviewer’s Responses to Questions

**Comments to the Author**

1. If the authors have adequately addressed your comments raised in a previous round of review and you feel that this manuscript is now acceptable for publication, you may indicate that here to bypass the “Comments to the Author” section, enter your conflict of interest statement in the “Confidential to Editor” section, and submit your "Accept" recommendation.

Reviewer #2: All comments have been addressed

Reviewer #3: All comments have been addressed

**********

2. Is the manuscript technically sound, and do the data support the conclusions?

The manuscript must describe a technically sound piece of scientific research with data that supports the conclusions. Experiments must have been conducted rigorously, with appropriate controls, replication, and sample sizes. The conclusions must be drawn appropriately based on the data presented.

Reviewer #2: Yes

Reviewer #3: Yes

**********

3. Has the statistical analysis been performed appropriately and rigorously?

Reviewer #2: N/A

Reviewer #3: Yes

**********

4. Have the authors made all data underlying the findings in their manuscript fully available?

The PLOS Data policy requires authors to make all data underlying the findings described in their manuscript fully available without restriction, with rare exception (please refer to the Data Availability Statement in the manuscript PDF file). The data should be provided as part of the manuscript or its supporting information, or deposited to a public repository. For example, in addition to summary statistics, the data points behind means, medians and variance measures should be available. If there are restrictions on publicly sharing data—e.g. participant privacy or use of data from a third party—those must be specified.

Reviewer #2: Yes

Reviewer #3: Yes

**********

5. Is the manuscript presented in an intelligible fashion and written in standard English?

PLOS ONE does not copyedit accepted manuscripts, so the language in submitted articles must be clear, correct, and unambiguous. Any typographical or grammatical errors should be corrected at revision, so please note any specific errors here.

Reviewer #2: Yes

Reviewer #3: Yes

**********

6. Review Comments to the Author

Please use the space provided to explain your answers to the questions above. You may also include additional comments for the author, including concerns about dual publication, research ethics, or publication ethics. (Please upload your review as an attachment if it exceeds 20,000 characters)

Reviewer #2: I would like to thank the authors for their careful and thoughtful responses to all reviewer comments. They have done an excellent job addressing the concerns raised during the review process, and the revisions have clearly strengthened the manuscript.

The revised manuscript is thorough, well-written, and coherent. The authors have provided adequate clarification where needed, and the overall presentation and flow of the paper have improved substantially. I appreciate the effort invested in revising the work and in engaging constructively with the reviewers’ feedback.

Overall, I am satisfied with the revisions and have no further comments. I believe the manuscript is now suitable for publication.

Reviewer #3: (No Response)

**********

7. PLOS authors have the option to publish the peer review history of their article (what does this mean?). If published, this will include your full peer review and any attached files.

If you choose “no”, your identity will remain anonymous but your review may still be made public.

**Do you want your identity to be public for this peer review?**  For information about this choice, including consent withdrawal, please see our Privacy Policy.

Reviewer #2: No

Reviewer #3: **Yes:** Leah I. Leinbach DMD MPH

**********

# Editor report: Acceptance letter

**DOI:** 10.1371/journal.pone.0352194.r006

**Authors:** Giang Vu, Giang Vu, Giang Vu

PONE-D-25-18997R2

PLOS One

Dear Dr. Attaran Kakhki,

I’m pleased to inform you that your manuscript has been deemed suitable for publication in PLOS One. Congratulations! Your manuscript is now being handed over to our production team.

At this stage, our production department will prepare your paper for publication. This includes ensuring the following:

* All references, tables, and figures are properly cited

* All relevant supporting information is included in the manuscript submission,

* There are no issues that prevent the paper from being properly typeset

You will receive further instructions from the production team, including instructions on how to review your proof when it is ready. Please keep in mind that we are working through a large volume of accepted articles, so please give us a few days to review your paper and let you know the next and final steps.

Lastly, if your institution or institutions have a press office, please let them know about your upcoming paper now to help maximize its impact. If they’ll be preparing press materials, please inform our press team within the next 48 hours. Your manuscript will remain under strict press embargo until 2 pm Eastern Time on the date of publication. For more information, please contact onepress@plos.org.

You will receive an invoice from PLOS for your publication fee after your manuscript has reached the completed accept phase. If you receive an email requesting payment before acceptance or for any other service, this may be a phishing scheme. Learn how to identify phishing emails and protect your accounts at https://explore.plos.org/phishing.

If we can help with anything else, please email us at customercare@plos.org.

Thank you for submitting your work to PLOS ONE and supporting open access.

Kind regards,

PLOS ONE Editorial Office Staff

on behalf of

Dr. Giang Truong Vu

Academic Editor

PLOS One
